# Scientometric Review for Research Patterns on Additive Manufacturing of Lattice Structures

**DOI:** 10.3390/ma15155323

**Published:** 2022-08-02

**Authors:** Chiemela Victor Amaechi, Emmanuel Folarin Adefuye, Irish Mpho Kgosiemang, Bo Huang, Ebube Charles Amaechi

**Affiliations:** 1 School of Engineering, Lancaster University, Bailrigg, Lancaster LA1 4YR, UK; 2 Standards Organisation of Nigeria (SON), 52 Lome Crescent, Wuse Zone 7, Abuja 900287, Federal Capital Territory, Nigeria; 3 Department of Mechanical/MetalWork Technology, Federal College of Education [Technical], Akoka 100001, Lagos State, Nigeria; 4 Department of Management, University of Central Lancashire (UCLAN), Preston PR1 2HE, UK; mikgosiemang1@uclan.ac.uk; 5 School of Civil Engineering, Hunan University of Science and Technology, Xiangtan 411201, China; bohuang@hnust.edu.cn; 6 Department of Zoology, University of Ilorin, Ilorin 240003, Kwara State, Nigeria; amaechi.ec@unilorin.edu.ng

**Keywords:** additive manufacturing, lattice structure, 3D printing, research pattern, research trend, scientometric, bibliometric, COVID19, scientific literature review, review, VOSviewer

## Abstract

Over the past 15 years, interest in additive manufacturing (AM) on lattice structures has significantly increased in producing 3D/4D objects. The purpose of this study is to gain a thorough grasp of the research pattern and the condition of the field’s research today as well as identify obstacles towards future research. To accomplish the purpose, this work undertakes a scientometric analysis of the international research conducted on additive manufacturing for lattice structure materials published from 2002 to 2022. A total of 1290 journal articles from the Web of Science (WoS) database and 1766 journal articles from the Scopus database were found using a search system. This paper applied scientometric science, which is based on bibliometric analysis. The data were subjected to a scientometric study, which looked at the number of publications, authorship, regions by countries, keyword co-occurrence, literature coupling, and scientometric mapping. VOSviewer was used to establish research patterns, visualize maps, and identify transcendental issues. Thus, the quantitative determination of the primary research framework, papers, and themes of this research field was possible. In order to shed light on current developments in additive manufacturing for lattice structures, an extensive systematic study is provided. The scientometric analysis revealed a strong bias towards researching AM on lattice structures but little concentration on technologies that emerge from it. It also outlined its unmet research needs, which can benefit both the industry and academia. This review makes a prediction for the future, with contributions by educating researchers, manufacturers, and other experts on the current state of AM for lattice structures.

## 1. Introduction

Over the past 15 years, interest in polymer additive manufacturing with several engineered materials has dramatically increased [1,2]. With numerous materials, this technique can be applied to quickly design and directly create three-dimensional (3D) objects without adding complexity to the production process. Recent studies into material developments include the microstructures, interfacial behaviour, pore density, layup patterns, layer thickness, and material development [3,4,5,6,7]. With the increasing need for better materials, there are increased techniques and technologies in material processing, materials developments, more customised materials, and newer engineered lattice-structured materials called additive manufactured materials [8,9,10,11,12,13,14]. Additive manufacturing (AM) has been practised for over 15 years in numerous manufacturing industries with the aid of 3D printing, referred to as additive manufacturing technology [15,16,17,18]. The medical, automotive, aerospace, and materials industries have all benefited from the innovation that additive manufacturing (AM) has brought forth [19,20,21,22]. It uses lithographic techniques to join materials, layer-by-layer, on top of an existing structure to create parts from 3D model data [23,24,25]. A variety of technologies, including rapid prototyping (RP), selective laser melting (SLM), and electron beam melting (EBM) technologies, are used in additive manufacturing (AM) [26,27,28,29]. The earlier technology, rapid prototyping, is a concept that refers to the rise of additive manufacturing (AM) and the development of the polymer material used for prototype [30]. One aspect of development of the models is the use of CAD (computer-aided design) to develop the AM designs, like on lattice structure prototyping. Contrary to conventional production methods like casting and machining, additive manufacturing enables designers to quickly prototype and reduce operational costs and material waste in the process [31,32,33]. For the process, two commonly used methods for building metal components from powder feedstock are the Directed Energy Deposition (DED) and Powder Bed Fusion (PBF) [34]. Another aspect of AM is the use of machine learning (ML), which has been proven in the optimization of the material properties, strength, material mix, and array of the lattice-structured additive manufactured materials [35,36,37].

**Figure 1:**
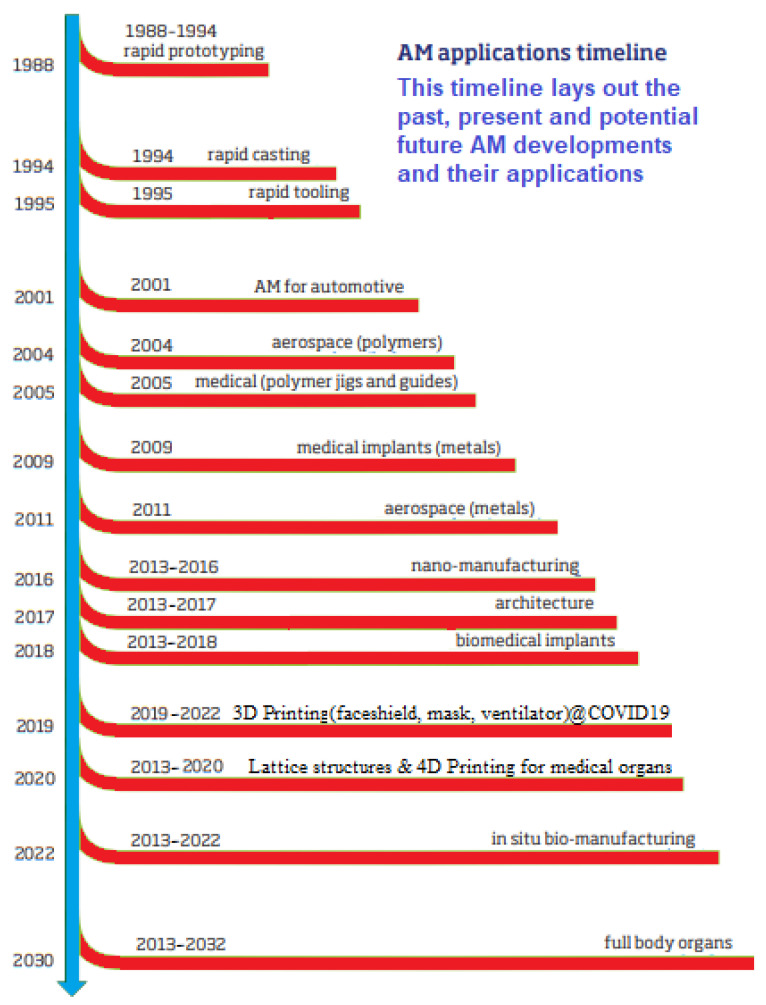
The timeline for the developments on additive manufacturing (AM), showing the past, present, and potential future (Adapted from original image with permission. Courtesy: Graham Tromans of Graham Tromans Associates, London, UK & Terry Wohlers of Wohlers Associates, Youngstown, OH, USA).

Earlier research using scientometric reviews on additive manufacturing reflect that there is an increase in research on other aspects of AM being investigated [38,39,40]. The current market demand places an ever-increasing emphasis on the efficient use of 3D printing for the production of complicated shapes [41,42,43]. However, the use of lattice structures in additive manufacturing has seen increasing demand due to their unique applications [44,45,46,47,48,49,50,51]. Lattice structures could be classified as porous and non-porous materials, depending on their applications [52,53,54,55,56,57,58,59,60]. These lattice structures for additive manufacturing have increasing applications from 3D printing to 4D printing, such as biological and medical applications [61,62,63,64,65,66,67,68]. Practical applications in the biomedical area that utilises lattice structures as additive manufactured materials include the manufacture of prosthetic legs and 3D-printed dental teeth. Conventional materials utilised for different advanced materials, such as ceramics, composites, or metals, can be found in additive manufactured (AM) materials [69,70,71]. However, AM has a method that spreads quickly in the manufacturing sectors, making AM products usable most of the time [72,73]. A 3D object scanner is used in additive manufacturing (AM), which enables the production of items with accurate geometrical details. In contrast to traditional manufacturing, which frequently necessitates milling or other processes to eliminate superfluous material, these are constructed layer by layer, much like a 3D printing process. There are also more experimental investigations on AM that are used with numerical investigations to further understand engineered lattice structures in AM [74,75,76]. Additionally, employing stereolithography (SL) for 3D systems, additive manufacturing (AM) technology solidified the thin UV (ultraviolet) layers with light-sensitive liquid polymer through laser operations. Additionally, additive manufacturing (AM) started to advance in the early 1980s when equipment was upgraded from a lower level of operation to a higher level using new conventional equipment as opposed to the previous equipment employed at the time, then in late 1980s to early 1990s, rapid prototyping increased [75,76]. These developments resulted from the use of more sophisticated jigs, medical implants, engineering applications, and tooling on the typical production floor, as earlier illustrated by Graham Tromans (UK) and Terry Wohler (USA) [75,76]. The timeline for the developments on AM showing the past, present, and potential future, including rapid casting (1994), rapid tooling (1995), AM for automotive (2001), aerospace polymers (2004), medical polymers and jigs (2005), medical metal implants (2009), aerospace polymers (2011), nano-manufacturing (2013–2016), architecture (2013–2017), biomedical implants (2013–2018), 3D printing of face shields, masks, ventilators during COVID19 pandemic (2019–2022), lattice structures and 4D Printing for medical organs (2013–2022), in situ bio-manufacturing (2013–2022), and full body organ printing (2013–2032), is represented in Figure 1.

These AM processes include material extrusion, material jetting, binder jetting, powder bed fusion, directed energy deposition, photopolymerization, and sheet lamination [76,77]. These processes are all used in additive manufacturing (AM) technologies. They are procedures and techniques for using parts produced through additive manufacturing in production facilities and public spaces. Some state-of-the-art reviews also present advantages of additive manufacturing with related bibliometric analysis, but they did not consider lattice structures [77,78,79]. Generally, the advantages of additive manufacturing (AM) technology include the use of complex geometries, lighter structures, and the material’s ability to allow customization. In addition, it allows manufacturing processes that involve an increase in the geometric complexity of the design or an increment of material volume that leads to a rise in the manufacturing cost or time, thus the need to improve upon this technology, as seen in the trends in both 3D printing and 4D printing [80,81,82,83,84,85,86]. Additionally, it is crucial to comprehend the current status of the literature in relation to additive manufacturing procedures and the mechanical properties of 3D printed materials. This technique has been used to produce 3D/4D objects without adding complexity to the production process. This understanding will help to establish a research horizon and create future works on this subject.

Hence the need to conduct this scientometric analysis on additive manufacturing for engineered lattice structures. The majority of the article is structured as follows: Section 1 introduces the subject area of AM. The research methodology is detailed in Section 2. The result and implications of the scientometric analysis are then addressed in Section 3. The implications of the systematic review with discussions on the research trends are detailed in Section 4. Lastly, the summary on the systematic review with recommendations for future research are presented in Section 5.

## 2. Materials and Methods

In this section, the materials used for the data analysis and the research methodology for the study are presented.

### 2.1. Data Retrieval

Data collection from the available literature was crucial to this study, notably for the scientometric analysis’s result. The data collection for this study followed the already established procedure for bibliometric reviews. Different studies on AM have covered a range of technologies applied [31,32,33,34,76,77,78,79], hence it is necessary to have a strategy for the selection of the papers. Two criteria were used in the literature collection strategy: (1) contemporary and relevance: all publications from 2002 to 2022 were searched, and the papers were manually screened by carefully reading the keywords and abstracts; and (2) quality assurance: only peer-reviewed papers from journals were included because journal papers typically go through careful reviews to remove errors and mistakes. For the literature review, the database choice was crucial. Due to its large coverage of the subject area in journal publications, the research repositories and academic databases were considered as knowledge domains. Thus, with the diverse range of academic databases cthat are presently available, there was need to decide on the choice of the database(s) to utilise. This decision was achieved by having an initial comparative study between the Scopus database and Web of Science (WoS), as both were considered to obtain the data, and they gave good results. The search was conducted using wildcards. The variations of one keyword were captured using the wildcard character *. The keywords chosen were (“additive manufacturing *” OR “lattice *” AND (“structure *”) based on the goal of this research. Searching for terms inside a publication’s title, abstract, or keywords turned up all of the available literature on multi-material additive manufacturing of polymers in the Scopus database. The 2002–2022 search window was chosen to reflect the current growth of polymer additive manufacturing using several materials. To restrict the number of papers published in peer-reviewed English journals, a screening procedure was used.

### 2.2. Research Methodology

**Figure 2:**
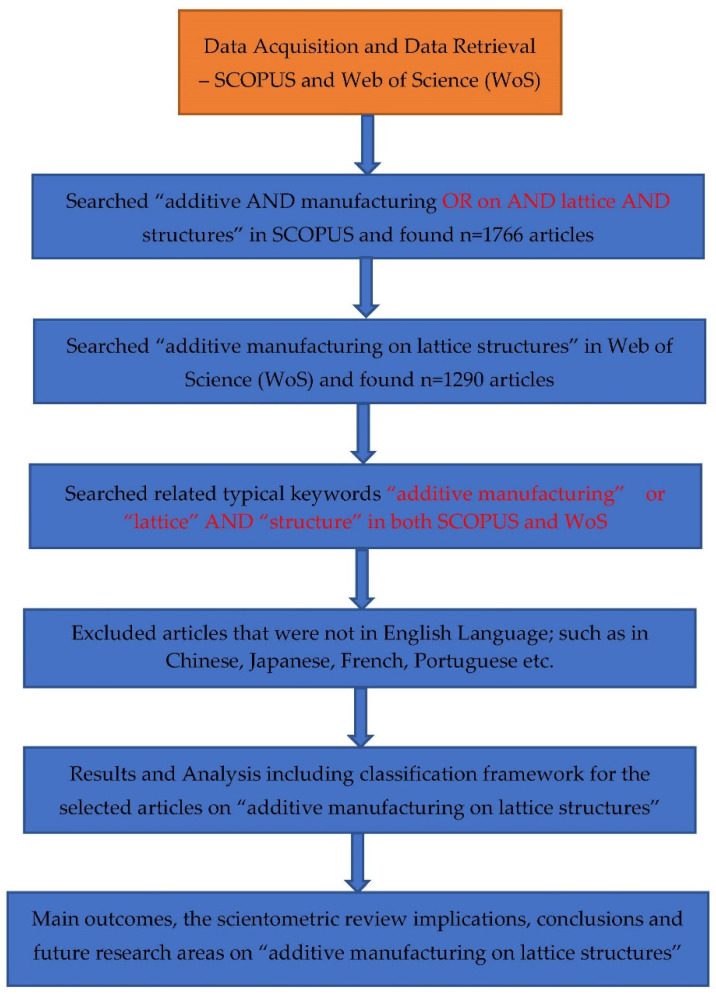
Research methodology on the scientometric study.

In this study, a scientometric analysis is carried out using research database and visualization-mapping tools to investigate research trends and patterns on the subject area. By inflection, scientometrics is used to reveal the research impact of publications, researchers, journals, and research institutions in a particular field of study. By definition, scientometrics also includes the quantitative study of science, science policy, and science communication, which gives an in-depth understanding of the research through the scientific citation and offers a deeper understanding of scientific citations [87,88,89]. To gain a thorough understanding of the evolution of this research field from 2002 to 2022, this study will conduct a scientometric evaluation and analysis of the papers pertaining to the multi-material additive manufacturing of engineered lattice structures. The scientometric review is also qualitatively validated by comparing the present data with other bibliometric studies on AM [38,39,40,90,91,92,93]. An extensive systematic review is then offered to offer deeper insights into the technology and applications of multi-material additive manufacturing of polymers based on the findings of the scientometrics analysis. In a nutshell, this study used a mixed review methodology, which combines scientometric analysis and systematic review, to examine the state of research on additive manufacturing for lattice-structure materials. By combining subjective research with a robust quantitative description and evaluation using science network mapping techniques, this study’s contribution can be seen as extending past review works in this field. The flowchart of the methodology is presented in Figure 2.

### 2.3. Article Selection

**Figure 3:**
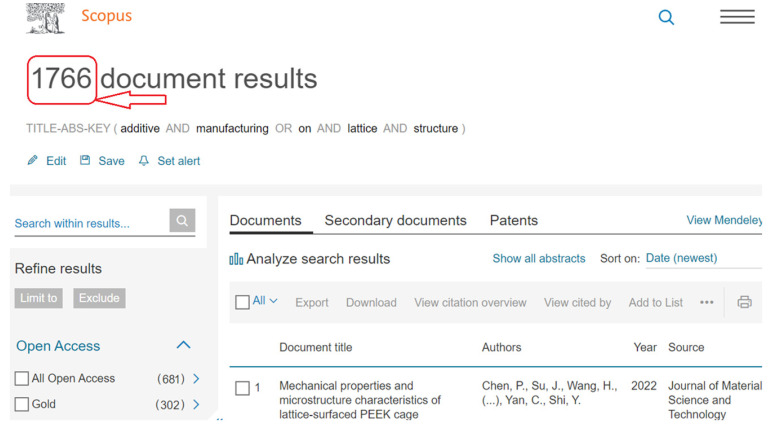
Scopus database supplied by Lancaster University UK, showing keyword “additive manufacturing on lattice structures” for meta-analysis (on 20 June 2022).

**Figure 4:**
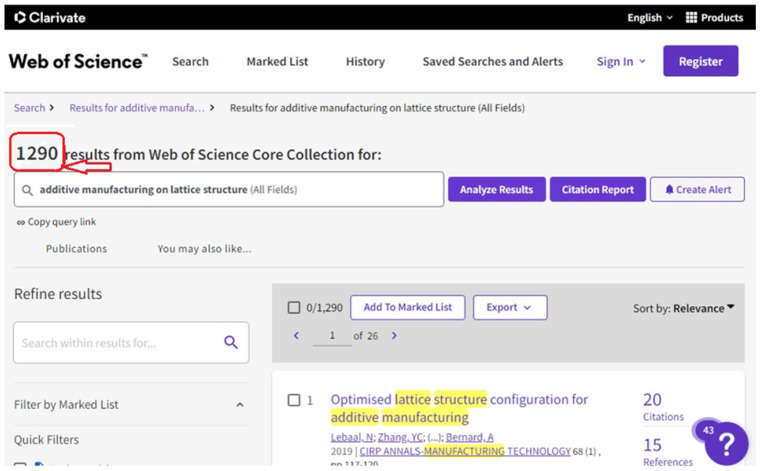
Web of Science (WoS) database supplied by Lancaster University UK, showing keyword “additive manufacturing on lattice structures” for meta-analysis (on 20 June 2022).

The method taken into account for choosing the academic papers is also a crucial component of the meta-science analysis carried out in this literature review. Finding research trends, threads, and advancements on additive manufacturing is one of the primary goals of this review. As represented in Figure 2, a public database named Scopus has been taken into consideration for this review in order to accomplish this objective. Scopus was accessed through Lancaster University, UK. After certain adjustments and exclusions to make sure the data used fits within the targeted study on additive manufacturing, a total number of papers were taken into account in the meta-analysis. Descriptors in the English language were taken from the Scopus database. Additionally, as the non-English papers were all disqualified, only English-language articles were taken into consideration. Clarification regarding the keywords that were used in this study are mentioned in the keyword search on Figure 2, Figure 3 and Figure 4, which show the databases used in this study.

Although some comparisons between data from the Scopus database (see Figure 3) and Web of Science database (see Figure 4) were done to determine the trend in development in other forms of the subject area, which remained the main keyword that the research was focused on, it should be noted that the representations in Figure 3 and Figure 4 were used to show different keyword searches used, as each database has a different search structure. Additionally, these database search images reflect that the search terms used in both show different lines but mean the same thing.

### 2.4. Research Indicators

The research indicators are key in identifying the significance of any research area. The influence of authorship, co-authorship, regions by countries, affiliations (or institutions), publication sources, and keywords are some of the aspects that are taken into account in the formulation of the scientometric investigation or similar bibliometric reviews [88,89,90,94,95,96,97,98,99,100,101,102]. Some mapping was conducted on the publication data retrieved using VoS Viewer [103,104,105,106,107,108,109,110,111,112], using standard methods of bibliometric mapping [113,114,115,116,117,118,119,120,121,122]. However, the publications were also screened by sampling some data, as the results were too much to check each paper. The trajectory of the analysed subject was tracked by measuring the impact factor and the h-index of the publications selected for the study. The impact factors of the published sources were found by scanning the Clarivate Analytics database [123], Web of Science (WoS) database [124], Scopus database [125], and the SCIMAGO database [126], also available in 2021 Journal Citation Reports [127]. Some studies investigated different databases ranging from PubMed to Scopus and Web of Science databases to conduct bibliometric analysis using different indicators [87,88,89,127,128,129,130]. However, as seen in Figure 3 and Figure 4, the search output obtained from Scopus were 1766 results, whereas the result from Web of Science (WoS) were 1290 results on the same keyword for this scientometric analysis. Hence, most of the data considered were from the Scopus database, whereas data from WoS was used to validate the studies. It also showed that Scopus had higher data collection on the subject area for the time range under consideration in this study. It should be noted that this does not reflect that one database has more collection of publication record than the other. Additionally, it should be noted that H-index is a particular indicator established by JE Hirsch in 2005, which measures each researcher’s number of publications and number of citations [128,129,131]. In that study, it was inferred that when a writer has N publications, and those publications have been mentioned at least N times by other writers, then that writer’s h-index is equal to N.

### 2.5. Scientometric Analysis

The knowledge domain structure of the multi-material additive manufacturing of polymers can be clarified by the discovered research clusters; however, the in-depth research problems and research demands cannot be revealed by scientometric analysis. In order to enhance the scientometric analysis in this work, a systematic review was carried out. The systematic review was first split into two parts by the authors: technology and applications. A consensus-based debate on the results of the scientometric review study led to the classification structure of research subjects in these two areas. It was reported that Nalimov and Mulchenko coined the word “Scientometrics” for the first time in 1969 as an evaluation of science [88,89,94,95]. From the second half of the 19th century to the present, scientometrics has been a growing field of study. In the past century, scientometrics research has progressed from the unconscious to consciousness, from qualitative to quantitative research, and from outward description to a thorough examination of the fundamental characteristics of scientific production. Recent research has shown the effectiveness of scientometrics in a variety of fields, including additive manufacturing [38,39,40], data analysis [94,95], built environment [96,97,128,129], research impact [131], sustainability [132,133], energy [134], project management [135], construction [136,137], water supply [138,139], medical applications [99,100,101,140,141], visualisation of data [103,104,105,106,107,108,114,115,116,117,118], and author collaborations [142,143,144]. Modern scientometric analysis enables researchers to access scientific contributions, map knowledge structures, to access scientific advancement, and identify emerging patterns within a certain study subject from these literature studies. It is quite difficult to describe the total field of multi-material additive manufacturing of polymers using simply systematic analysis due to the large range of research subjects that fall under this umbrella [145,146]. The research field can be understood in depth through systematic analysis, but this method has limitations in terms of subjective interpretation and is open to bias [134,135,136]. In order to analyse the findings of earlier studies in the field of multi-material additive manufacturing of polymers, a scientometrics analysis method was proposed in this work.

### 2.6. VOS Viewer

**Figure 5:**
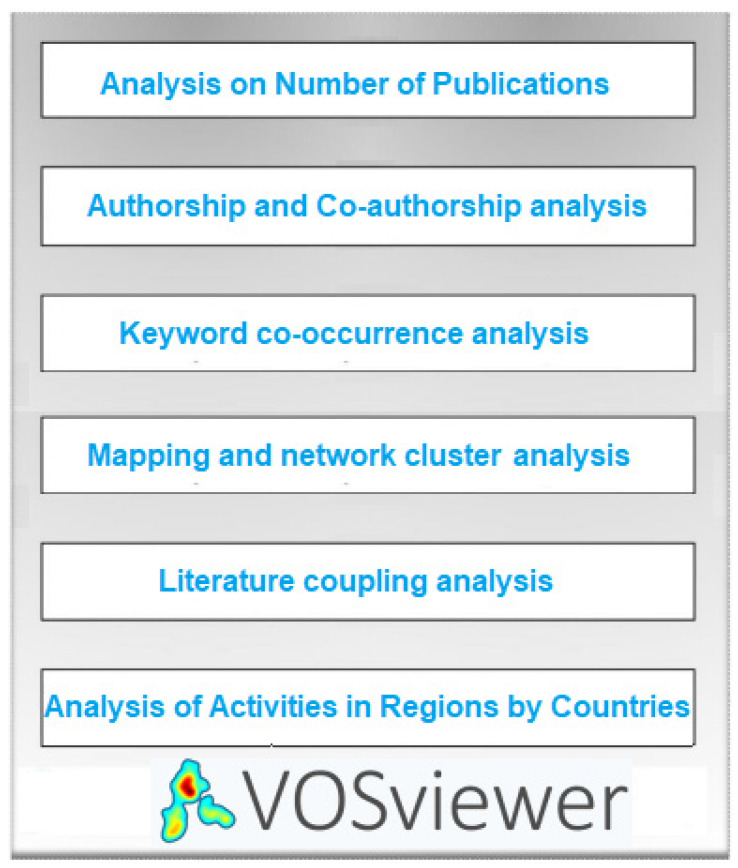
Different analyses conducted using VOSviewer.

The VOSviewer is an open-source programme and was used in this study’s network modelling and visualisation [103,104,105,106,107,108]. Nees Jan van Eck and Ludo Waltman currently own the VOSviewer. For this study, the version of the software used is VOSviewer version 1.6.18, and it was run with Java version 1.8.0_333 and Microsoft Graph. It is important to note that when organising research subjects for the ensuing systematic review, both the scientific mapping of research communities and themes derived from literature coupling analysis and keyword co-occurrence analysis were taken into account. Numerous analyses were conducted from different angles, including analyses of the countries/regions’ activity, authorship, co-occurrence, keyword, literature coupling, and number of publications, as summarised in Figure 5.

## 3. Results and Analysis

### 3.1. Publication History

**Figure 6:**
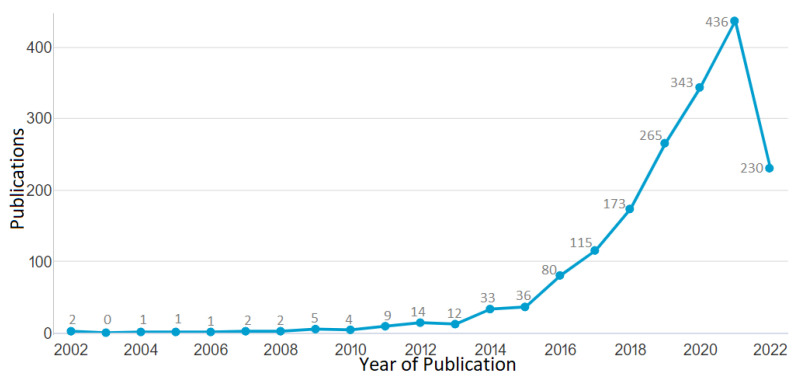
The number of publications against years of publication for publications from 2002 to 2022 (data retrieved from the Scopus database on 20 June 2022).

The first aspect of the results for the component meta-analysis is the impact of the research and its breakdown of publication years. Data from the Scopus database was obtained on 20 June 2022, as shown in Figure 6. From the result, the publishing output from 2015–2021 showed a modest trend shift when the most recent articles were taken into account. The output increased from 33 publications in 2014, to 36 publications in 2015, to 80 publications in 2016, before it reduced to 115 publications in 2017. Then it increased to 173 publications in 2018, went up to 265 publications in 2019, increased to 343 publications in 2020, peaked at 436 publications in 2021 while they were producing materials to control the Corona Virus, then decreased to 230 publications by the middle of 2022. As a result, among other things, it may be said that the research is a function of economic activity, as 1766 journal papers were published between 2002 and 2022 using the literature search technique described in Section 3. Figure 6 displays the annual number of journal publications on the subject of additive manufacturing. This statistic shows a general rising trend from 2006 to 2009. Starting in 2010, a burst was noticeable because there were only four publications, which can be ascribed to the global economic crisis. From there, it increased dramatically until the year 2021, rising to nine publications in 2011 and fourteen publications in 2012. The number of publications increased at an astounding rate between 2013 and 2021. Notably, the surge that began in 2013 coincided with an important development in additive manufacturing technology, also summarised in Figure 1. The particular developments seen in recent times from Figure 1 are seen in nano-manufacturing, architecture, engineering of car body parts like brake pedals, engineering of COVID19 control devices like ventilators, personal protective equipment (PPE) like face shields, biomedical implants like prosthetic bones, in situ bio-manufacturing, and full body organs. The growing accessibility of established additive manufacturing technologies may be responsible for the rise in study into the additive manufacture of designed lattice structures in recent years.

### 3.2. Publication Sources

**Figure 7:**
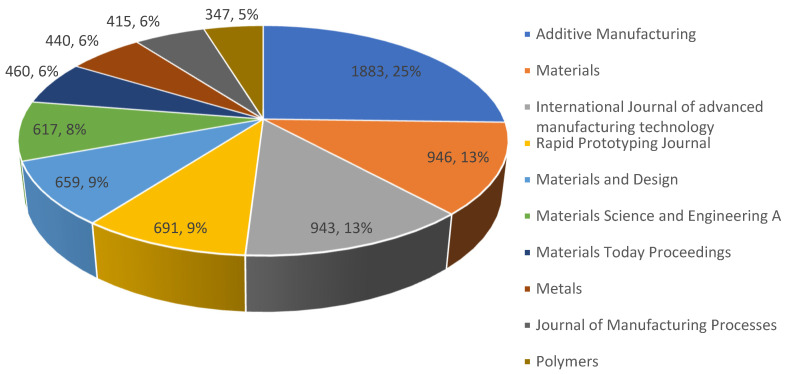
The total number of publications per year by source against percentage of publications from 2001 to 2022 (data retrieved from the Scopus database on 20 June 2022). Note: Each segment shows the number of publications and the corresponding percentages, which is separated by a comma (,) for each publication source.

**Figure 8:**
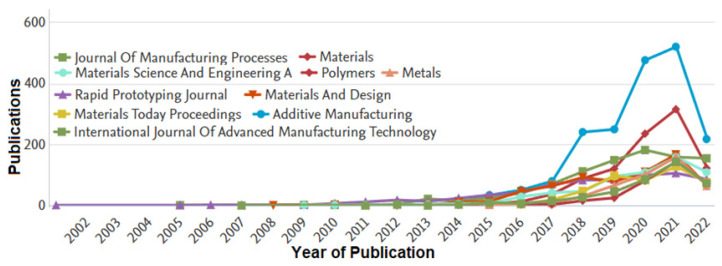
The number of publications per year by source against years of publication for publications from 2001 to 2022 (data retrieved from the Scopus database on 20 June 2022).

The publishing sources are the subject of the current meta-analysis. Other academic databases were searched as well, though, to verify the information from the Scopus database. It was decided to use the Scopus, despite considering major academic databases like PubMed, Science Direct, DOAJ, Web of Science, Google Scholar, and Scopus. In a detailed, methodical, and scientometric review of scientific scholarly articles (or papers) from journals and conferences, it was possible to make more inferences on the investigation of additive manufacturing. Academic publishers with academic repositories and databases, such as Taylor & Francis, Elsevier, Sage, and Springer Link, were also taken into account, as seen in Figure 7 and Figure 8. Journals that had high significance are specialist journals like Additive Manufacturing, which has a high h-index with an impact factor of 10 and citescore of 11.60, as well as international conferences like ASME, ASCE, ICE, ICCM, ICCS, NIST, ICCS, SAMPE, ISOPE, OTC, etc., were also taken into consideration. As can be seen in Figure 7, the majority of publications on AM were presented in journal papers from two important conference proceedings. However, these publications were less numerous than those that appeared in related Q1 journals. The outcome was subsequently vetted to include the best journals in additive manufacturing. Elsevier’s Additive Manufacturing, Elsevier’s Materials and Design, MDPI’s Materials, MDPI’s Polymers, and MDPI’s Metals were the journals that appeared the most frequently. The other periodicals are International Journal of Advanced Manufacturing Technology, Rapid Prototyping Journal, Materials Today Proceedings, and Journal of Manufacturing Processes. This was further analysed in Table 1 to show that the highest data was published in Additive Manufacturing journal, especially from the years 2018–2022, where they have high marginal increase.

Further analysis of the publications per year by source was conducted on this area using data from Web of Science (WoS). It was observed that many publications were available that generally researched on additive manufacturing from 2002–2022. From the data obtained from Scopus, Additive Manufacturing published 1883 articles, Materials published 946 articles, International Journal of advanced manufacturing technology published 940 articles, Rapid Prototyping Journal published 683 articles, Materials and Design published 658 articles, Materials Science and Engineering A published 616 articles, Materials Today Proceedings published 460 articles, Metals published 440 articles, Journal of Manufacturing Processes published 415 articles, and Polymers published 347 articles. However, the sourcing of the data was also conducted using the WoS (Web of Science) database. From the WoS database, 46,821 articles were retrieved, whereas the Scopus database had 43,602 articles. The survey reveals that all of the papers’ research output grew from 2013, but Elsevier’s Additive Manufacturing had the highest publishing rate. This demonstrates that additive manufacturing researchers have encountered similar problems. These problems primarily revolve around the mechanics of materials with lattice structures, the number of layers, the thickness of the lattice, the material compositions, and the development of standards for lattice structure additive manufacturing. Secondly, it was discovered that, between 2002 and 2022, many patents were published by various inventors as a result of the earliest increased developments in lattice-structured additive produced materials, which were noted as early as in 2002.

### 3.3. Publication Subjects

**Figure 9:**
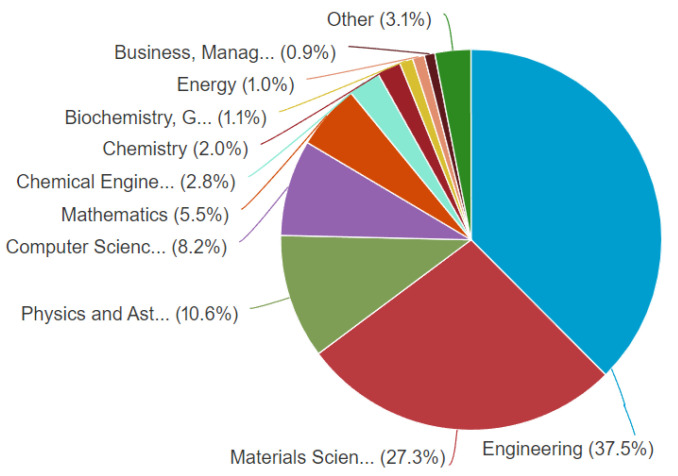
Literature search distribution on the classification of publications by subjects on ‘additive manufacturing on lattice structures’, (Scopus database on 20 June 2022).

**Figure 10:**
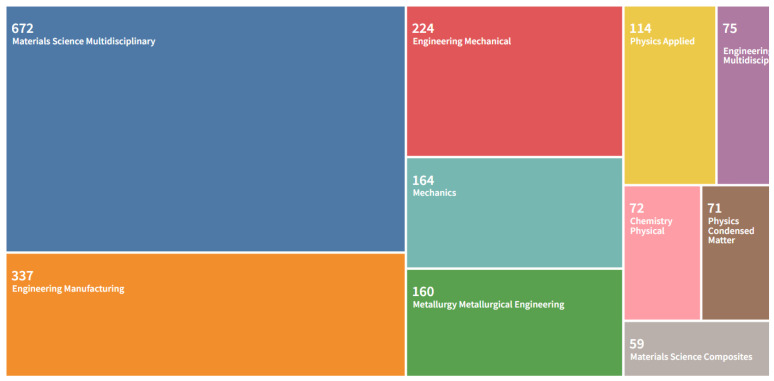
Visualisation treemap chart of different disciplines that published articles on “additive manufacturing on lattice structure” research (data retrieved from the WoS database on 20 June 2022).

The meta-analysis conducted on the scientometric review in this section focuses on the literature search using publication subjects as presented in Figure 9 and Figure 10. They represent the subject-based categorization of papers on additive manufacturing for engineered lattice structures. Engineering-related disciplines accounted for the largest percentage in the 2022 data at 37.5%, followed by Materials Sciences at 27.1%; these two occupied over 50% of the quadrat on publication subjects. It was followed by Physics and Astronomy at 10.6%, then Computer Sciences at 8.2%, then Mathematics at 5.5%, then Chemical Engineering at 2.8%, then Chemistry at 2.0%, then Biochemistry at 1.1%, then Energy at 1.0%. The least was achieved by Business Management at 0.9%, whereas Others, which included minor subgroups, were at 3.1%, which showed that there were other evolving areas that worked on application of additive manufacturing. Furthermore, it was noted that research on Engineering in 2022 data surpassed other areas, which could be seen in the need to develop control materials for the COVID-19 pandemic and systems for manufacturing and the production of oil and gas, among others. These are seen in some of the sampled papers from the screening conducted on the papers used in this study. In other comparable domains, similar transitions were seen using Web of Science data (see Table 2). The data on Table 2 were used to give the best significance of the study, as it has been unified and approximated to be 3 s.f. (significant figures). The tabulated data were also used to have a breakdown of different engineering subjects, such as Engineering Manufacturing, Engineering Mechanical, and Engineering Multidisciplinary. The visualisation treemap used for all publications on additive manufacturing showed that Materials Science Multidisciplinary had 672 publications, followed by Engineering Manufacturing at 337 publications, followed by Engineering Mechanical at 224 publications, followed by Mechanics at 164 publications, followed by Engineering Metallurgy at 160 publications, followed by Applied Physics at 114 publications, followed by Engineering Multidisciplinary at 75 publications, followed by Physical Chemistry at 72 publications, followed by Condensed Matter Physics at 71 publications, and the least was Materials Science Composites at 59 publications. This further demonstrates how interest on additive manufactured materials in engineering subjects has been influenced by their use in full-scale applications, control systems for the COVID-19 pandemic (like face shields), pipeline fabrication, fabrication of machine parts, and deployment on cutting-edge systems. 

### 3.4. Publication Type

**Figure 11:**
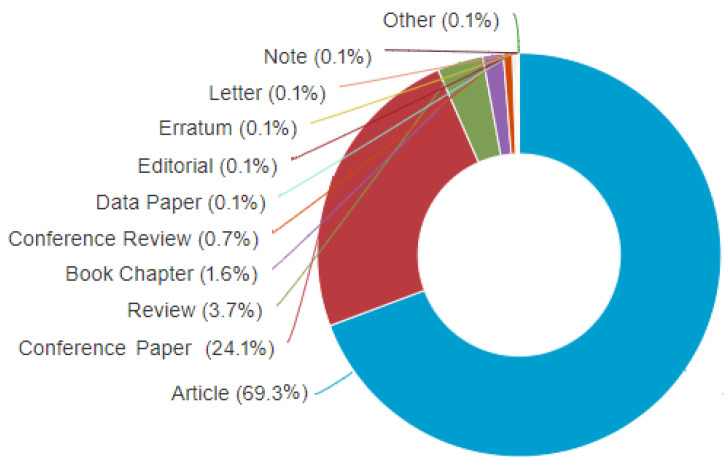
Literature search distribution on the classification of publication type on ‘additive manufacturing on lattice structures’, (Scopus database on 20 June 2022).

The meta-analysis conducted on the scientometric review in this section focuses on the literature search using publication type that is presented in Figure 11. It represents the type-based categorization of papers on additive manufacturing for lattice structures. Journal papers (or articles) are seen to be the highest, with 69.3% having 1226 publications, followed by conference papers, at 24.1% having 427 publications. Next are review papers, at 3.7% producing 65 documents, then book chapters, at 1.6% producing 29 documents, followed by conference review, at 0.7% producing 13 documents. The other types including the notes, letters, errata, editorials, and data papers; each produced 0.1%, reflecting two documents from each type. This implies that the research scrutiny on AM is reflected on the volume of publication outputs, which are significantly research articles.

### 3.5. Publication Keywords

**Figure 12:**
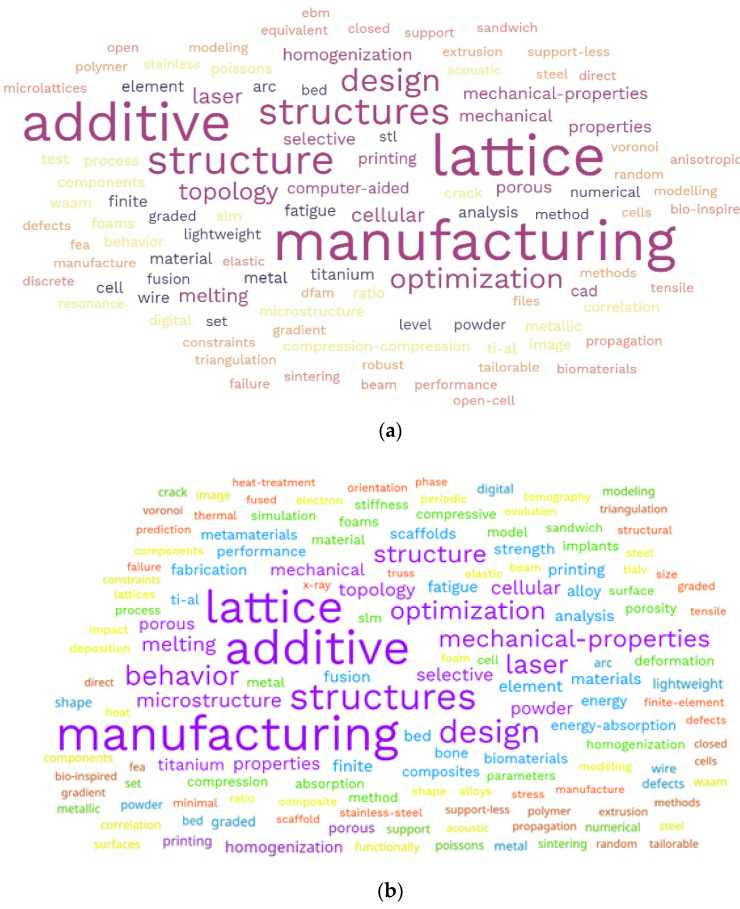
Word cloud on keywords published articles on “additive manufacturing on lattice structure” research (data retrieved from WoS database on 20 June 2022), showing (**a**) scheme 1 and (**b**) scheme 2.

The scientometic analysis on the publication keywords on the search keywords on this investigation. This investigation was initially conducted on the keywords using word cloud, which showed that some words had higher density than others, as seen in Figure 12. The densest keywords are represented with higher font sizes and unique font colours. The keywords are visualized in order using a word cloud generator, which shows the highest to the lowest as boldest to the least bold. The keywords include lattice, structures, additive, manufacturing, design, behaviour, mechanical-properties, optimization, laser, microstructure, structure, melting, mechanical, topology, porous, melting, powder, etc. The word cloud was developed, using text mining via an online Free Word Cloud Generator, to generate two schemes of a word cloud based on different amounts of keywords, as seen in Figure 12a,b. The lesser the number of keywords, the smaller the form of the word cloud, as seen in Figure 12a. However, when more keywords were used, the limit of the word cloud generator had to be increased to develop Figure 12b, but the limit was 100 words. It was then compared with another generator called Voyant tool, which had much larger limit of up to 500 words.

**Figure 13:**
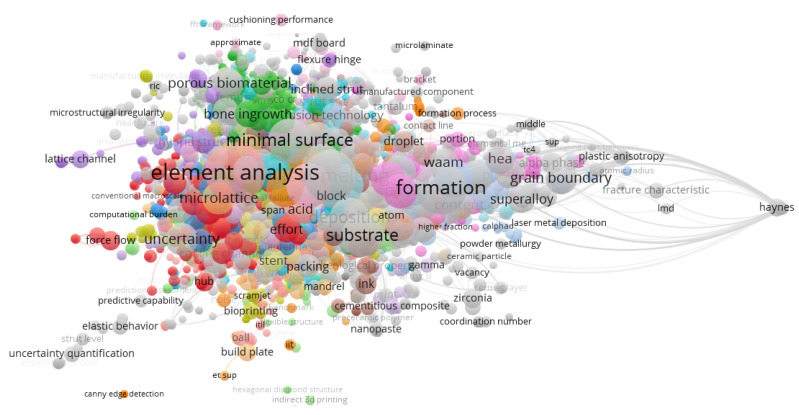
Visualization of a network for keywords published in articles on “additive manufacturing on lattice structure” research (data retrieved from the WoS database on 20 June 2022 and visualized with VOSviewer).

**Figure 14:**
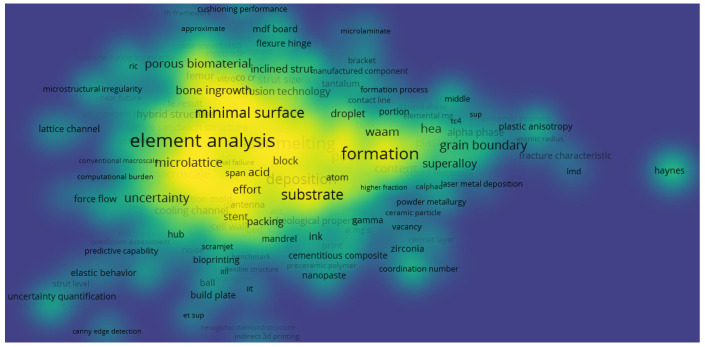
Bibliometric map showing a visualization of the density for keywords published in articles on “additive manufacturing on lattice structure” research (data retrieved from the WoS database on 20 June 2022 and visualized via VOSviewer).

However, the keywords from the scientometric analysis were further post-processed, using VOSviewer version 1.6.18, to obtain the network visualization and density visualization in Figure 13 and Figure 14. The mapped networks showed 36 clusters, showing the co-occurrences of bibliometric items used for the keywords. From this search, there were 3920 items from the results for the component meta-analysis. It was observed that the highest keyword co-occurrence was “element analysis”, which shows that a lot of work on this area has been considered based on the different designs for lattice structures used in additive manufacturing. The type of element used has an impact on the research by increasing more micropores, microstructures, and element analysis of the lattices used for the breakdown of publication years from 2002 to 2022. Other keywords that make a mark on this area are: formation, microlattice, minimal surface, porous biomaterial, FE (finite element) result, etc. These range show the diverse research conducted within the scope of additive manufacturing on lattice structures. See the supplementary data for the keyword files used in developing the word clouds and other aspects of this scientometric review.

### 3.6. Publication Affiliation

**Figure 15:**
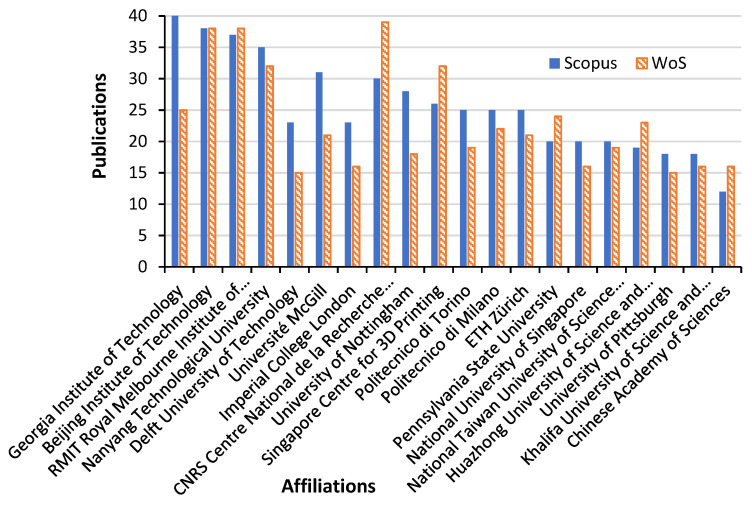
Publication affiliation for “additive manufacturing on lattice structure” research (data retrieved from Scopus and WoS on 20 June 2022).

**Figure 16:**
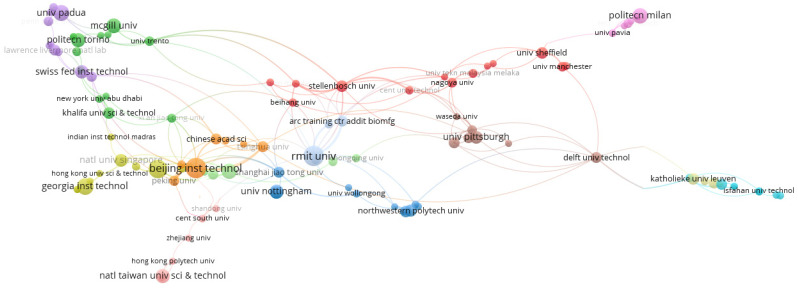
Visualization of network for affiliations on “additive manufacturing on lattice structure” research (data retrieved from WoS database on 20 June 2022 and visualized with VOS Viewer).

This sub-section presents the results of the publication affiliation from the bibliometric analysis on the subject area. The results of research output related to the publication in this field are important in understanding the research patterns and the impact of affiliations (institutions and organisations), on the research. A deeper understanding of the support from different affiliations to additive manufacturing on lattice structures is necessary to assess the research impact from the institution or organisation, which is given as a breakdown of publication volume from different departments. Moreso, applications of additive manufacturing on lattice structures have been seen in bioengineering, medical applications, and mechanical engineering. Hence, the outputs seen from the databases were cross-field publications. Currently, different research institutes, polytechnics, universities, and companies have contributed to the scientific literature on additive manufacturing on lattice-manufactured materials. However, there was a recent increase in small-scale research and small AM businesses during the recent COVID-19 pandemic, as detailed in Section 4. AM applications were seen in the control of CoronaVirus for the production of PPEs like face shields, and also in fabricating ventilators [9]. To better understand the influence of affiliations, the analysis of publication affiliations was conducted using data from SCOPUS and WoS databases. To visualise the mapped network, the author’s names were further filtered to see publications produced on this subject area per year. This also helps to see the impact of the institution on the research strength in that area. Figure 15 shows the affiliation contributions on the subject matter. There are over 160 institutions that have contributed to research in AM on lattice structures. Each of these institutions have different authors, and some of the publications are sponsored or funded by different funders. However, further analysis on the impact of the funding agencies is presented in the next sub-section. The affiliations have collection of documents, as well as some have only one publication, as AM on lattice structures is still developing in some institutions. Table 3 presents the twenty (20) institutions with the highest publications on the subject area. From this study’s data, the Georgia Institute of Technology had the highest publications, as it produced 40 publications on Scopus database while 25 publications on WoS database. The affiliation with the second highest publication is Beijing Institute of Technology which produced 38 publications on both Scopus and WoS databases. The next affiliation is Royal Melbourne Institute of Technology (RMIT) as it produced 37 publications on Scopus database while 38 publications on WoS database. It was also observed that the publications from these top affiliations were also published in high impact journals. Also, the publications from these highest affiliations are among those who have had many years of research experience on the subject area on additive manufacturing. Lastly, the visualized network map in Figure 16 also shows the research connectivity of the different affiliations with 13 clusters.

### 3.7. Publication Authors

**Figure 17:**
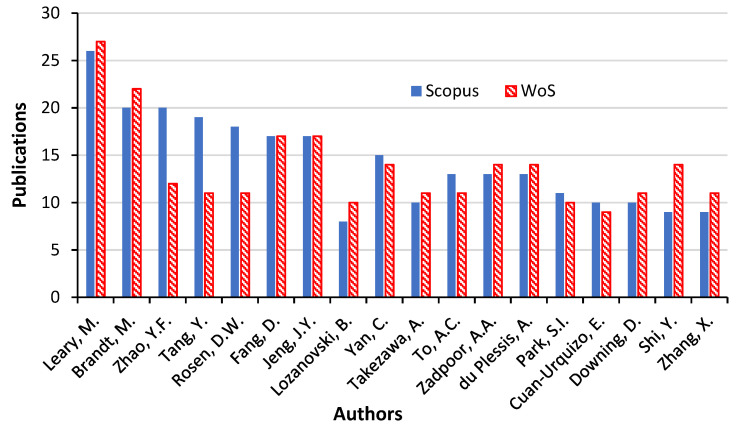
Publication authors for “additive manufacturing on lattice structure” research (data retrieved from Scopus and WoS on 20 June 2022).

Another aspect of the investigation of research patterns on “additive manufacturing on lattice structures” is based on the publication authorship. The first aspect of the component meta-analysis is understanding the impact of authorship on the research and its breakdown of publication volume. Different researchers have contributed to the scientific literature on additive manufacturing on lattice-manufactured materials. To visualise the mapped network, the author’s names were further filtered to see publications that did not have more than 25 authors per publication. Figure 17 shows the authorship contributions on the subject matter. There are over 2000 authors in the collection of documents, and some of them have only one publication. Table 4 presents the eighteen authors with the highest h-index and highest publications. Additionally, the year the documents were published is shown. The total number of citations since they first published documents and the quantity of references cited for each work were retrieved from academic databases. From this study’s data, the author with the highest h-index is Leary, M., followed by Brandt, M., and then next is Zhao, Y.F. The authors who received the most citations per publication were also examined in more detail in the next section. It should be noted that the latest works by authors with the highest h-index are the contributions of the most widely referenced related work. However, authors with the highest h-index are among those who have had many years of research experience on the subject area on additive manufacturing.

### 3.8. Publication Citations

**Figure 18:**
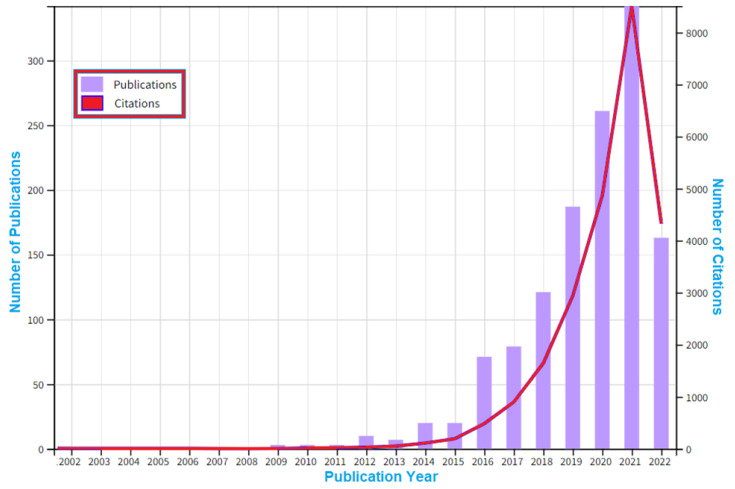
Number of citations and number of publications from 2002 to 2022 for “additive manufacturing on lattice structure” research (data retrieved from WoS on 20 June 2022).

In this sub-section, the scientometric review on publication citations is conducted. The citations in this field against the quantity of publications is one important factor to consider. The citations are used to assess the strength of a research area, the scientific significance, and the impact of the publications in the subject area. One of the indicators used in this assessment is the h-index. The h-index value is based on a list of publications ranked in descending order by the Times Cited count. It can be said that an index of ‘h’ implies that there are ‘h’ papers that have each been cited at least ‘h’ times. Additionally, the h-index is based on the depth of years of the WoS database product subscription and your selected timespan. The source items that are not part of the WoS database product subscription were not factored into the calculation. There were less publications from WoS, whereas there were more publications in Scopus, in a ratio of 1766:1294. It was observed that there was a h-index of 79 and an average citation per publication of 18.71. In the total documents on the subject area, there were also 12,691 citing articles, whereby 11,750 publications were without self-citations. These articles were cited 24,205 times, cumulatively, whereas those publications without self-citations were cited 19,028 times. This is shown in the citation data presented in Figure 18. The number of documents has increased significantly since 2013, whereas the slope of the cumulative publications has barely changed. With the exception of a little decline in the 2012–2013 era, the most substantial changes in the slope of the cumulative publications are shown between 2009–2021. It is important to note there has not been a plateau pattern in recent years, which shows that additive manufacturing research for lattice structures has been relevant recently. Additionally, the drop in the 2021–2022 data shows a drop because it is mid-2022; as such, it is expected to tip higher. 

### 3.9. Publication Collaborations by Co-Authorship

**Figure 19:**
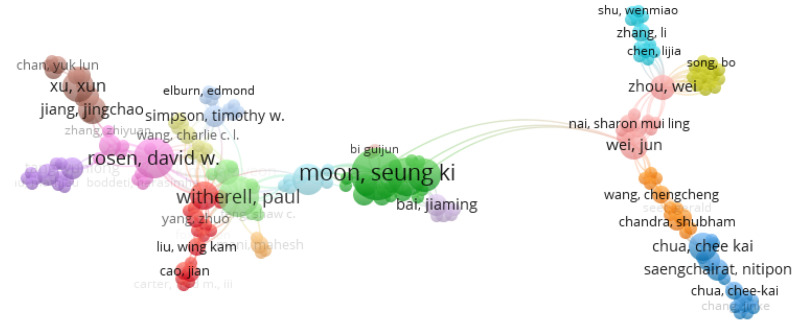
Mapping based on co-authorship showing the network visualization for the first method.

**Figure 20:**
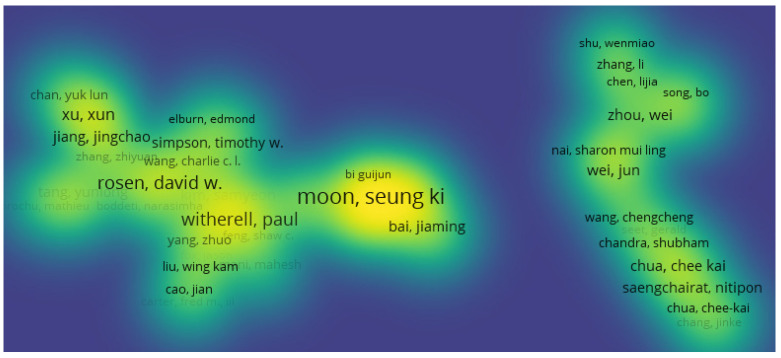
Mapping based on co-authorship showing the density visualization for the first method.

The scientometric analysis on publication authorship was conducted using the data from the publication databases. For the co-authorship analysis, the counting method used was the full counting method, and the publications that had above 25 authors were ignored from this study. The number of documents per author were limited to five, and 92 met the thresholds out of 4475 authors. The authors with the greatest total link strength were used in the selection. The threshold system used to filter the authorship was a minimum of one publication in the area, as 3446 authors met this threshold. In this data clustering, there were two methods considered in the analysis. For the first method, 17 clusters were used for the authorship, as seen in Figure 19 and Figure 20. The highest publications were identified in the green node by cluster 2 showing Seung Ki Moon as the most published author in additive manufacturing, with 23 links, 17 publications, and a total link strength of 45.

**Figure 21:**
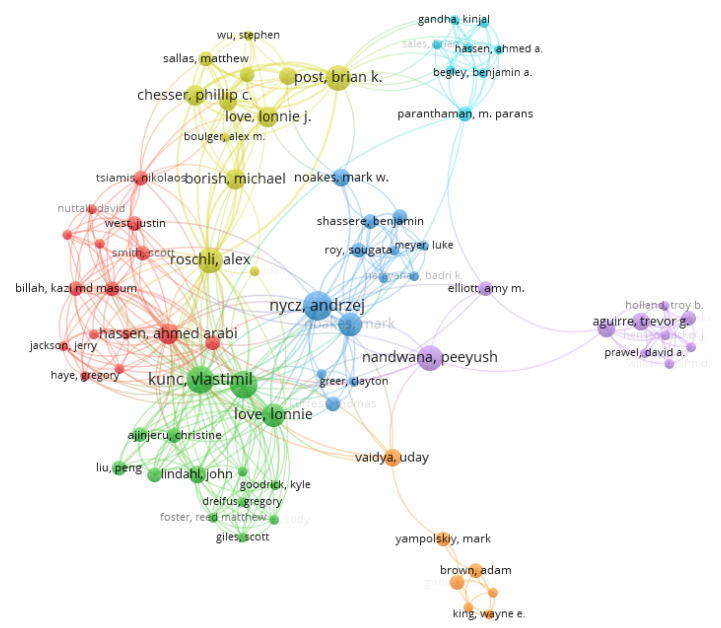
Mapping based on co-authorship showing the network visualization for the second method.

**Figure 22:**
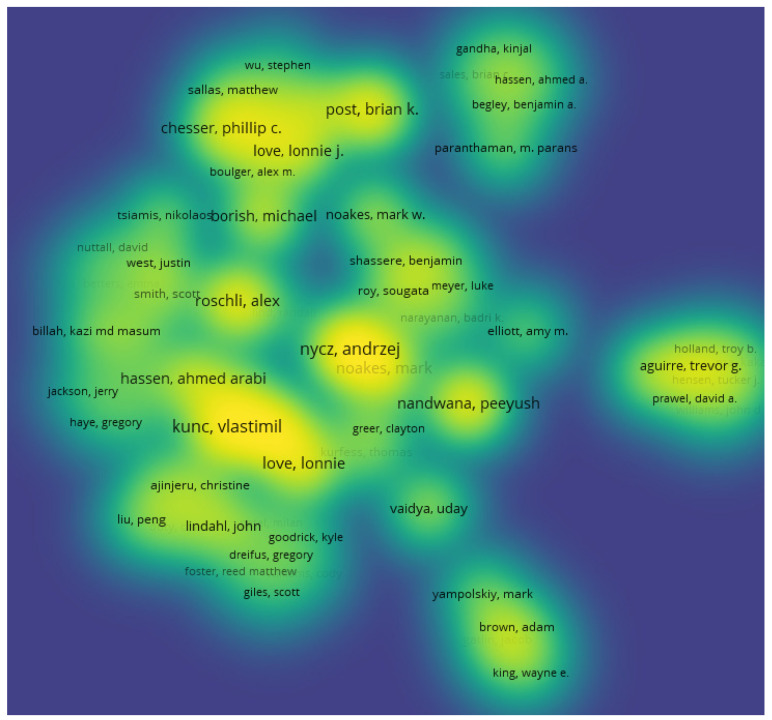
Mapping based on co-authorship showing the density visualization for the second method.

The second method of analysis was conducted using fractionalization to normalize the data. It showed a much wider network of co-occurrences between publications but mapped more links and clusters between the different authors in different locations, as seen in Figure 21. This method also showed the impact of the research, as seen through the authors. The density visualization in Figure 22 also showed the link strength of the authors on this subject area, and the breakdown of publication can be tracked to see the research patterns.

### 3.10. Publication Countries/Regions

**Figure 23:**
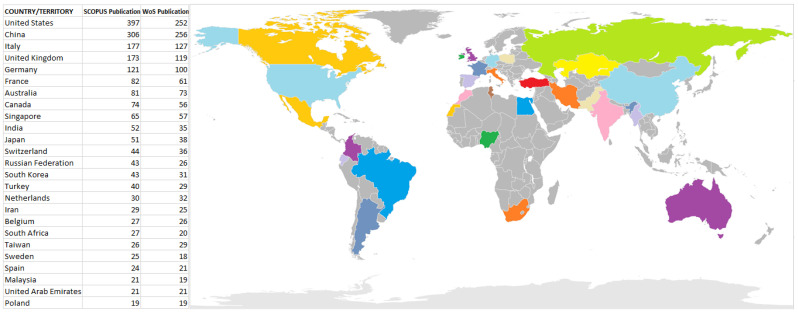
The global research activities showing countries with the highest relevance for “additive manufacturing on lattice structures”.

The scientometric analysis on publications conducted in this subject area showed that the researchers from 75 different nations have contributed to the scientific literature on additive manufacturing on lattice structure materials, although only 25 of those nations have more than 10 publications to their names. Figure 23 shows the 25 nations with the most quantity of publications, namely: United States, China, Italy, United Kingdom, Germany, France, Australia, Canada, Singapore, India, Japan, Switzerland, Russian Federation, South Korea, Turkey, Netherlands, Iran, Belgium, South Africa, Taiwan, Sweden, Spain, Malaysia, United Arab Emirates, and Poland. According to the overall number of publications that are not dependent on international collaboration, the USA comes out as the top nation. From 2002–2022, it was observed that different databases reflected close results for each country, as seen in Table 5. The Scopus database showed that the USA produced 397 publications, whereas WoS showed that the USA produced 252 publications. The top four countries with the highest scientific production also include Italy, China, and the UK. In this way, it can also be seen that the regions of Europe and Asia, where there are more than 60 publications, are more interested in research on additive manufacturing with lattice structures. As shown in Figure 23, the USA, China, Italy and the UK, which have the broadest worldwide network of collaboration, are at the forefront of academic engagement. It is hoped that other nations will close the gap in the publication ratio from that of the top countries, such as USA, which almost doubles the third (Italy).

## 4. Implications of Trends for Future Research

### 4.1. Implications of Publication Volume

Due to the significant advantages that polymer-based materials have recently brought to the research and industrial community globally, new studies and technological developments have centred on enhancing levels of multifunctionality in diverse applications. When compared to single homogenous structures, the ability to fabricate bespoke multi-material structures utilising additive manufacturing technology enabled particular material selection and improved various attributes [145,146,147,148,149,150]. Considering the nature of the topics covered in this review, more discussion has been extended to the limitations of “additive manufacturing on lattice structures” and the challenges of including more aspects of the bibliometric analysis. Hence, further discussions should be looked at based on three (3) very relevant aspects: “type of additive manufacturing technology”, “lattice topologies”, and “additive manufacturing”. Based on the scientometric analysis, the following evaluation of recent multi-material polymers with AM applications in the engineering, biomedical, and information technology sectors is provided.

**Figure 24:**
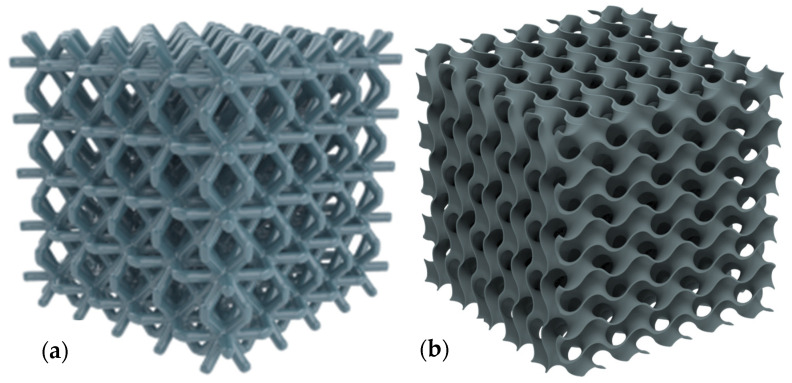
Typical additive manufactured representations showing the computational model (**a**) a strut-based lattice structure, and (**b**) a surface-based lattice structure.

The literature on lattice-structured materials manufactured using additive processes has grown significantly since 2002 up to 2022, as seen in Figure 6. This pattern not only reflects the advancement of additive manufacturing technology, but it also reflects the rising need for further research. There were over 10 unique high-impact journals that publish in a widely diversified set of publications, which were presented in Table 1. Although journal publications are evenly distributed, the field of additive manufacturing on lattice structures has the most publications overall at 25% (Additive Manufacturing Journal). The field of additive manufacturing, which encompasses a variety of applications, systems, methodologies, techniques, materials, and technologies, is often regarded as having the top journal in the world. Table 1 indicates that the majority of the journals, with the exception of Additive Manufacturing and Rapid Prototyping Journal, concentrate on materials. Therefore, in the process of determining where to publish their papers, researchers working on technologies or processes of additive manufacturing may run into problems. Some of these additive manufacturing studies include different types of lattice structures [150,151,152,153,154,155,156,157,158,159]. Typical representations showing the computational model for typical lattice structures, such as (a) the strut-based lattice structure, and (b) the surface-based lattice structure, are seen in Figure 24.

### 4.2. Implications of Additive Manufacturing Processes

The correlations between keywords in articles were taken into account in this analysis. Different studies show that the mechanical properties of both polymer and metal material additive manufacturing received the most attention from researchers [160,161,162,163,164,165,166,167,168]. Although important for new functional polymers, the applications of fire-resistance, electrical, thermal, bioprinting, electronics, 4D printing, and biocompatible qualities garnered far less research in this discipline. Different processes for AM, including related terminologies on AM, can be seen in the ISO standard [169]. Fused deposition modelling (FDM), which is thought to be the most popular method for multi-material additive manufacturing due to its expanding choice of materials and doable technique, is another hot place from the keywords network. The problems with the FDM technology of additive manufacturing, particularly the weak bond strength between various materials, have not yet been solved, and both academia and business should pay greater attention to this issue.

The purpose of the AM research on lattice structures is to advance 3D/4D printing techniques that fill the gaps between this technology and conventional production processes. Currently, the creation of polymeric compounds opens the door to the flexibility to examine and regulate the characteristics and functionality of the product manufacturing process that are relevant to their actual application. To enhance the quality of the finished product in accordance with its geometry, research into the development of novel materials and investigation of the physical processes involved during the deposition process must be combined. The researcher will be able to determine the composition of the microstructures and the mechanical properties based on the chosen printing parameters. Additionally, future research can be done to evaluate the behaviour during the development of numerical models to assess the temperature variation and the thermal stress sustained by the material.

The scientometric analysis conducted in this study was able to examine different parameters of publications on the field. However, the research findings were only able to quantitatively determine any prospective research gaps and probable future trends. Based on new areas to look into, one possible topic for research in the realm of additive manufacturing is printing efficiency. It is frequently necessary to make a trade-off between printing effectiveness (such as scanning speed) and part quality (such as print resolution) [38,39,40,41]. In order to increase printing efficiency, more energy power or quicker scanning speeds can be used, although printing accuracy may suffer as a result. Additionally, lengthy, complicated post-processes lengthen the printing process overall. It is also challenging to expand the printing or production platform, since there are post-processing problems such as with heat treatment and support material removal. Therefore, it is essential to continuously create and enhance efficient post-processing techniques.

### 4.3. Implications of Policy Documents 

The implication of policy documents on the subject area were also looked at based on the scientometric analysis conducted in this study. However, it is suggested that an in-depth analysis be conducted in this area, as policies related to AM are evolving due to the material advances made, developments of techniques for AM, applications, global challenges, and economic values. More study is recommended on the elaboration of AM standards to cover both 3D printing and 4D printing. Additionally, since there are other forms of lattice structures like the planar lattice structure, octet lattice structure, and the BCC lattice structure, it is pertinent that more specialised bibliometric analysis will be conducted that would be confined to particular forms of lattice structures. However, the present scientometric analysis was able to examine different parameters of publications on the field. Additionally, the research findings were only able to quantitatively determine these prospective research gaps and probable future trends.

Another constraint on lattice architectures is the interfacial bonding strength. Engineering the interfaces between objects made of different materials presents one of the challenges in multi-material 3D printing. Determining the proper level of connections for the lattice structure is therefore necessary. Even though different additive manufacturing techniques have made significant advancements and have a great deal of room for growth in the future, the weak bond strength between adjacent printed layers of various materials remains a challenge. This challenge is due to the formation of defects brought on by variations in the physical and chemical properties of the materials, which would ultimately affect the overall mechanical performance of the printed parts [38,39,40].

Lattice structures benefit from the optimization of additive manufacturing. There are various methods for utilising this solution to improve printing parameters. To get over this problem, this can be achieved using prototype fabrication, numerical simulation, in-situ monitoring, or artificial intelligence. Additionally, anisotropy in the printed object may result from the use of many materials in 3D printing, and each layer’s mechanical characteristics may decline as a result of the manufacturing process’ temperature gradient. There are two main categories of methods for increasing the mechanical strength of multi-material 3D parts: processing parameter optimization [149,158,159] and additional external energy input [147,148,156]. To push the limits of multi-material additive manufacturing, fundamental scientific understanding on the inter-layer cohesion mechanisms between incompatible printed materials is required, in addition to technical innovations.

### 4.4. Implications of the COVID-19 Pandemic

Thus, it is possible to develop more unique materials and applications for this field. An application of response from this field also induces the publication history as recorded in Section 3.1. Two global events have been considered in the publication trend, as the 2016–2017 oil price decline and the COVID-19 pandemic have been highlighted. It is clear from the 2022 data that the sizes are compared to one another. Additionally, it illustrates the impact of several events, such as the COVID-19 pandemic and the global decline in the price of oil per barrel in the years 2016–2017. The authors propose that these two worldwide events have an impact on the volume of publications for additive manufacturing on lattice structures, especially COVID-19 [169,170,171,172,173,174,175,176,177,178,179]. The production of high-caliber research articles has been significantly influenced by the recent worldwide COVID-19 epidemic in 2020–2021 and national lockdowns that happened in over 80% of countries globally for many months due to the challenge of finding COVID-19 controls such as the use of 3D printed face shields [9,120,180,181,182,183,184,185,186,187,188]. Also, lessons learnt from the recent COVID19 pandemic has shown that challenging situations can lead to advances in AM [189,190].

Although the majority of commercial 3D printers can create macro-scale parts, there are several real-world uses for 3D printed parts at various scales. A promising printing technology for 3D printing parts from the micro to nanoscale of various materials appears to be the hybrid 3D printing platform, which can balance resolution and printing efficiency at the micro-nanometer scale. The next part provides a summary of the future directions that the systematic review suggests, with concluding remarks that are based on the authors’ expertise.

## 5. Conclusions

With the increasing designs and advances in material development, additive manufacturing has not been left behind. Additive manufacturing has aided the manufacture of 3D parts and other multi-material polymer/metal additive manufactured components. Thus, the need for this scientometric study to investigate the research patterns for additive manufacturing on lattice structures. In-depth assessments were conducted on the literature in the area by looking at publication records from the Scopus and WoS databases. The primary research frameworks, articles, and pertinent research subjects were subsequently discovered through analysis of the number of publications, literature coupling, keyword co-occurrence, authorship, and countries/regions activities.

This paper offers a thorough, systematic analysis of the most recent developments in additive manufacturing, both in terms of technique and applications for publications from 2002 to 2022. The scientometric analysis revealed a strong bias in favour of investigating materials in this area but little concentration on emerging technologies. The author keywords from this bibliometric review shows that there are different aspects of lattice structure that are related to the subject area. For instance, 3D printing efficiency, interfacial bonding strength between multiple materials, cross-contamination, scalability, and applications stated above are just a few of the lingering issues that additive manufacturing technology still faces today, seen in the keywords.

To overcome these obstacles, interdisciplinary research and development will be crucial, and developments in additive manufacturing on lattice structures and its innovative uses in new fields will hasten scientific research and technical advancement in a variety of fields. VOSviewer was also used to visualize the items, the co-relationships of the clusters, and the mapped networks needed. This was achieved by using a consistent data format to retrieve the data from both databases. From this study, the USA was the highest region that worked on additive manufacturing for lattice structures. This research also shows patterns of research from authors and their affiliations.

It is recommended that the data from one database be validated by using a second database and comparing the publication records. To gain more complete data, it can be improved by merging several datasets like Scopus, Google Scholar, and Web of Science. Additionally, the scientometric analysis in this study is unable to directly offer or depict the expertise of researchers or publication authors, which may prevent further review. Further areas of scientific reviews can be involved with technical focus groups, whereby the publications can be grouped into many categories. Additionally, the results of the scientometric analysis in each category can be sent to a corresponding expert from technical focus groups. This approach can be used to retrieve technical views from experts in various fields. Findings from the plethora of literature on AM can further present advancements made in the field and the research gaps to broaden the scope of recommendations.

## Figures and Tables

**Table 1:** Publications in Scopus in the top journals for additive manufacturing on lattice structures.

Publication Source	Scopus								
	Total Publications (TP)	2021 Articles	2020 Articles	2019 Articles	2018 Articles	2017 Articles	CiteScore	SJR	SNIP
Additive Manufacturing	1883	521	477	250	241	80	11.6	2.71	2.946
Materials	946	316	236	121	89	37	4.2	0.682	1.261
International Journal of advanced manufacturing technology	943	159	182	149	112	71	5.6	0.946	1.486
Rapid Prototyping Journal	691	106	100	85	83	70	6.0	0.827	1.281
Materials and Design	659	168	111	78	93	64	13.0	1.842	2.264
Materials Science and Engineering A	617	159	109	96	47	42	8.8	1.574	1.973
Materials Today Proceedings	460	128	83	98	48	19	1.8	0.341	0.657
Metals	440	160	102	66	31	8	3.4	0.57	1.062
Journal of Manufacturing Processes	415	144	86	45	27	15	6.6	1.387	2.084
Polymers	347	144	82	25	16	3	4.7	0.77	1.2

**Table 2:** Data on publication subjects on “additive manufacturing on lattice structure” from WoS.

Web of Science Categories	Record Count	% of 1294
Materials Science Multidisciplinary	672	51.932
Engineering Manufacturing	337	26.043
Engineering Mechanical	224	17.311
Mechanics	164	12.674
Metallurgy Metallurgical Engineering	160	12.365
Physics Applied	114	8.81
Engineering Multidisciplinary	75	5.796
Chemistry Physical	72	5.564
Physics Condensed Matter	71	5.487
Materials Science Composites	59	4.56
Engineering Biomedical	56	4.328
Materials Science Biomaterials	48	3.709
Nanoscience Nanotechnology	48	3.709
Automation Control Systems	46	3.555
Materials Science Characterization Testing	42	3.246
Engineering Industrial	35	2.705
Computer Science Interdisciplinary Applications	29	2.241
Mathematics Interdisciplinary Applications	26	2.009
Thermodynamics	26	2.009
Engineering Electrical Electronic	25	1.932
Chemistry Multidisciplinary	22	1.7
Engineering Civil	21	1.623
Instruments Instrumentation	21	1.623
Multidisciplinary Sciences	21	1.623
Polymer Science	21	1.623

**Table 3:** Comparisons on publication affiliation for “additive manufacturing on lattice structure” research (data retrieved from Scopus and WoS on 20 June 2022).

Affiliation	Scopus	WoS	% of 513	% of 465
Georgia Institute of Technology	40	25	7.797	5.376
Beijing Institute of Technology	38	38	7.407	8.172
RMIT Royal Melbourne Institute of Technology	37	38	7.212	8.172
Nanyang Technological University	35	32	6.823	6.882
Delft University of Technology	23	15	4.483	3.226
Université McGill	31	21	6.043	4.516
Imperial College London	23	16	4.483	3.441
CNRS Centre National de la Recherche Scientifique	30	39	5.848	8.387
University of Nottingham	28	18	5.458	3.871
Singapore Centre for 3D Printing	26	32	5.068	6.882
Politecnico di Torino	25	19	4.873	4.086
Politecnico di Milano	25	22	4.873	4.731
ETH Zürich	25	21	4.873	4.516
Pennsylvania State University	20	24	3.899	5.161
National University of Singapore	20	16	3.899	3.441
National Taiwan University of Science and Technology	20	19	3.899	4.086
Huazhong University of Science and Technology	19	23	3.704	4.946
University of Pittsburgh	18	15	3.509	3.226
Khalifa University of Science and Technology	18	16	3.509	3.441
Chinese Academy of Sciences	12	16	2.339	3.441

**Table 4:** Comparative results on publication authors for “additive manufacturing on lattice structure” research (data retrieved from Scopus and WoS on 20 June 2022).

Author Name	Scopus	WoS	% of 258	% of 246
Leary, M.	26	27	10.078	10.976
Brandt, M.	20	22	7.752	8.943
Zhao, Y.F.	20	12	7.752	4.878
Tang, Y.	19	11	7.364	4.472
Rosen, D.W.	18	11	6.977	4.472
Fang, D.	17	17	6.589	6.911
Jeng, J.Y.	17	17	6.589	6.911
Lozanovski, B.	8	10	3.101	4.065
Yan, C.	15	14	5.814	5.691
Takezawa, A.	10	11	3.876	4.472
To, A.C.	13	11	5.039	4.472
Zadpoor, A.A.	13	14	5.039	5.691
du Plessis, A.	13	14	5.039	5.691
Park, S.I.	11	10	4.264	4.065
Cuan-Urquizo, E.	10	9	3.876	3.659
Downing, D.	10	11	3.876	4.472
Shi, Y.	9	14	3.488	5.691
Zhang, X.	9	11	3.488	4.472

**Table 5:** The global research activities showing countries with the highest relevance for “additive manufacturing on lattice structures” from the Scopus and WoS databases.

Country/Territory	Scopus	WoS	% of Scopus Sum (1998)	% of WoS Sum (1526)
United States	397	252	19.869	16.514
China	306	256	15.315	16.776
Italy	177	127	8.859	8.322
United Kingdom	173	119	8.659	7.798
Germany	121	100	6.056	6.553
France	82	61	4.104	3.997
Australia	81	73	4.054	4.784
Canada	74	56	3.704	3.669
Singapore	65	57	3.253	3.735
India	52	35	2.603	2.294
Japan	51	38	2.553	2.490
Switzerland	44	36	2.202	2.359
Russian Federation	43	26	2.152	1.704
South Korea	43	31	2.152	2.031
Turkey	40	29	2.002	1.900
Netherlands	30	32	1.502	2.097
Iran	29	25	1.451	1.638
Belgium	27	26	1.351	1.704
South Africa	27	20	1.351	1.311
Taiwan	26	29	1.301	1.900
Sweden	25	18	1.251	1.180
Spain	24	21	1.201	1.376
Malaysia	21	19	1.051	1.245
United Arab Emirates	21	21	1.051	1.376
Poland	19	19	0.951	1.245

## Data Availability

The raw/processed data required to reproduce these findings have been shared along with supplementary data as used on the present study.

## Supplementary Materials

The following supporting information can be downloaded at: Amaechi, Chiemela Victor; Adefuye, Emmanuel (2022), “Supplementary Dataset on Scientometrics of Additive Manufacturing for lattice structure”, Mendeley Data, V1, https://doi.org/10.17632/wxd98kfkrp.1 (accessed on 22 July 2022).

## References

[1] Cheng B., Chou K. (2015). Geometric consideration of support structures in part overhang fabrications by electron beam additive manufacturing. Comput. Aided Des..

[2] Bui T.Q., Hu X. (2021). A review of phase-field models, fundamentals and their applications to composite laminates. Eng. Fract. Mech..

[3] Amaechi C.V., Gillet N., Odijie A.C., Hou X., Ye J. (2019). Composite Risers for Deep Waters Using a Numerical Modelling Approach. Compos. Struct..

[4] Ye J., Cai H., Liu L., Zhai Z., Amaechi C.V., Wang Y., Wan L., Yang D., Chen X., Ye J. (2021). Microscale intrinsic properties of hybrid unidirectional/woven composite laminates: Part I: Experimental tests. Compos. Struct..

[5] Amaechi C.V., Gillet N., Ja’e I.A., Wang C. (2022). Tailoring the local design of deep water composite risers to minimise structural weight. J. Compos. Sci..

[6] Blok G., Longana L., Yu H., Woods S. (2018). An investigation into 3D printing of fibre reinforced thermoplastic composites. Addit. Manuf..

[7] Amaechi C.V. (2022). Local tailored design of deep water composite risers subjected to burst, collapse and tension loads. Ocean. Eng..

[8] Mirzendehdel A.M., Suresh K. (2016). Support structure constrained topology optimization for additive manufacturing. Comput. Aided Des..

[9] Advincula R., Dizon J., Chen Q., Niu I., Chung J., Kilpatrick L., Newman R. (2020). Additive manufacturing for COVID-19: Devices, materials, prospects, and challenges. MRS Commun..

[10] Hussein A., Hao L., Yan C. (2013). Advanced Lattice Support Structures for Metal Additive Manufacturing. J. Mater. Process. Technol..

[11] Ranjan R., Samant R., Anand S. Design for Manufacturability in Additive Manufacturing Using a Graph Based Approach. Proceedings of the ASME 2015 International Manufacturing Science and Engineering Conference.

[12] Vayre B., Vignat F., Villeneuve F. (2012). Designing for Additive Manufacturing. Procedia CIRP.

[13] Dong G., Tessier D., Zhao Y.F. (2019). Design of Shoe Soles Using Lattice Structures Fabricated by Additive Manufacturing. Proceedings of the Design Society: International Conference on Engineering Design.

[14] Diegel O., Schutte J., Ferreira A., Chan Y.L. (2020). Design for additive manufacturing process for a lightweight hydraulic manifold. Addit. Manuf..

[15] Dar U.A., Mian H.H., Abid M., Topa A., Sheikh M.Z., Bilal M. (2020). Experimental and numerical investigation of compressive behavior of lattice structures manufactured through projection micro stereolithography. Mater. Today Commun..

[16] Xie G., Dong Y., Zhou J., Sheng Z. (2020). Topology optimization design of hydraulic valve blocks for additive manufacturing. Proc. Inst. Mech. Eng. Part C J. Mech. Eng. Sci..

[17] Cheng L., To A. (2019). Part-scale build orientation optimization for minimizing residual stress and support volume for metal additive manufacturing: Theory and experimental validation. Comput. Aided Des..

[18] Cheng L., Liang X., Bai J., Chen Q., To J.L.A. (2019). On utilizing topology optimization to design support structure to prevent residual stress induced build failure in laser powder bed metal additive manufacturing. Addit. Manuf..

[19] Salmi M. (2021). Additive Manufacturing Processes in Medical Applications. Materials.

[20] Zhang K., Qu H., Guan H., Zhang J., Zhang X., Xie X., Yan L., Wang C. (2021). Design and Fabrication Technology of Metal Mirrors Based on Additive Manufacturing: A Review. Appl. Sci..

[21] Wang X., Wang C., Zhou X., Wang D., Zhang M., Gao Y., Wang L., Zhang P. (2020). Evaluating Lattice Mechanical Properties for Lightweight Heat-Resistant Load-Bearing Structure Design. Materials.

[22] Ning F., Cong W., Jia Z., Wang F., Zhang M. Additive manufacturing of CFRP composites using fused deposition modeling: Effects of process parameters 1989. Proceedings of the ASME 2016 11th International Manufacturing Science and Engineering Conference.

[23] Korshunova N., Alaimo G., Hosseini S.B., Carraturo M., Reali A., Niiranen J., Auricchio F., Rank E., Kollmannsberger S. (2021). Bending behavior of octet-truss lattice structures: Modelling options, numerical characterization and experimental validation. Mater. Des..

[24] Azzouz L., Chen Y., Zarrelli M., Pearce J.M., Mitchell L., Ren G., Grasso M. (2019). Mechanical properties of 3-D printed truss-like lattice biopolymer non-stochastic structures for sandwich panels with natural fibre composite skins. Compos. Struct..

[25] Long J., Nand A., Ray S. (2021). Application of Spectroscopy in Additive Manufacturing. Materials.

[26] Obadimu S.O., Kourousis K.I. (2021). Compressive Behaviour of Additively Manufactured Lattice Structures: A Review. Aerospace.

[27] Ueno A., Guo H., Takezawa A., Moritoyo R., Kitamura M. (2021). Temperature Distribution Design Based on Variable Lattice Density Optimization and Metal Additive Manufacturing. Symmetry.

[28] Huang J., Zhang Q., Scarpa F., Liu Y., Leng J. (2016). Bending and benchmark of zero Poisson’s ratio cellular structures. Compos. Struct..

[29] Chowdhury S., Mhapsekar K., Anand S. (2018). Part Build Orientation Optimization and Neural Network-Based Geometry Compensation for Additive Manufacturing Process. J. Manuf. Sci. Eng. Trans. ASME.

[30] Choi S., Samavedam S. (2002). Modelling and Optimisation of Rapid Prototyping. Comput. Ind..

[31] Stichel T., Laumer T., Linnenweber T., Amend P., Roth S. (2016). Mass flow characterization of selective deposition of polymer powders with vibrating nozzles for laser beam melting of multi-material components. Phys. Procedia.

[32] Chianrabutra S., Mellor B.G., Yang S., Bourell D.L. (2014). A Dry Powder Material Delivery Device for Multiple Material Additive Manufacturing. Proceedings of the 25th Annual International Solid Freeform Fabrication Symposium: An Additive Manufacturing Conference.

[33] Kruth J.P., Mercelis P., Van Vaerenbergh J., Froyen L., Rombouts M. (2005). Binding mechanisms in selective laser sintering and selective laser melting. Rapid Prototyp. J..

[34] Babuska Tomas F., Krick Brandon A., Susan Donald F., Kustas Andrew B. (2021). Comparison of powder bed fusion and directed energy deposition for tailoring mechanical properties of traditionally brittle alloys. Manuf. Lett..

[35] Grierson D., Rennie A.E.W., Quayle S.D. (2021). Machine Learning for Additive Manufacturing. Encyclopedia.

[36] Wang C., Tan X.P., Tor S.B., Lim C.S. (2020). Machine learning in additive manufacturing: State-of-the-art and perspectives. Addit. Manuf..

[37] Zhang Y., Dong G., Yang S., Zhao Y.F. Machine learning assisted prediction of the manufacturability of laser-based powder bed fusion process. Proceedings of the ASME Design Engineering Technical Conference; American Society of Mechanical Engineers (ASME).

[38] Zheng Y., Zhang W., Baca Lopez D.M., Ahmad R. (2021). Scientometric Analysis and Systematic Review of Multi-Material Additive Manufacturing of Polymers. Polymers.

[39] García-León R.A., Gómez-Camperos J.A., Jaramillo H.Y. (2021). Scientometric Review of Trends on the Mechanical Properties of Additive Manufacturing and 3D Printing. J. Mater. Eng. Perform..

[40] Jin Y., Ji S., Li X., Yu J. (2017). A scientometric review of hotspots and emerging trends in additive manufacturing. J. Manuf. Technol. Manag..

[41] Osama S., Al-Ahmari Ameen W., Mian S. (2019). 2019 Additive manufacturing: Challenge, Trends, and Applications. Adv. Mech. Eng..

[42] Chabaud G., Castro M., Denoual C., Le Duigou A. (2019). Hygromechanical properties of 3D printed continuous carbon and glass fibre reinforced polyamide composite for outdoor structural applications *Addit*. Manuf..

[43] Liao G., Li Z., Cheng Y., Xu D., Zhu D., Jiang S., Guo J., Chen X., Xu G., Zhu Y. (2018). Properties of oriented carbon fiber/polyamide 12 composite parts fabricated by fused deposition modeling. Mater. Des..

[44] Wauthle R., Vrancken B., Beynaerts B., Jorissen K., Schrooten J., Kruth J.P., Van Humbeeck J. (2015). Effects of build orientation and heat treatment on the microstructure and mechanical properties of selective laser melted Ti6Al4V lattice structures. Addit. Manuf..

[45] Gorny B., Niendorf T., Lackmann J., Thoene M., Troester T., Maier H.J. (2011). In situ characterization of the deformation and failure behaviour of non-stochastic porous structures processed by selective laser melting. Mater. Sci. Eng. A.

[46] Mohsenizadeh M., Gasbarri F., Munther M., Beheshti A., Davami K. (2018). Additively manufactured lightweight metamaterials for energy absorption. Mater. Des..

[47] Maconachie T., Leary M., Lozanovski B., Zhang X., Qian M., Faruque O., Brandt M. (2019). 2019 SLM lattice structures: Properties, performance, applications, and challenges. Mater. Des..

[48] Mark C. (2016). Optimal lattice-structured materials. J. Mech. Phys. Solids.

[49] Mona M., Methods for Modelling Lattice Structures (2020). Kth Royal Institute of Technology School of Engineering Sciences. Master’s Thesis.

[50] Pan C., Han Y., Lu J. (2020). Design and optimization of lattice structures: A review. Appl. Sci..

[51] Rosen D.W., Johnston S.R., Reed M. Design of general lattice structures for lightweight and compliance applications. Proceedings of the Rapid Manufacturing Conference.

[52] Ashby M.F. (2006). The properties of foams and lattices. Philos. Trans. R. Soc. A Math. Phys. Eng. Sci..

[53] Gibson L.J., Ashby M.F. (1997). Cellular Solids: Structure and Properties.

[54] Meza L.R., Das S., Greer J.R. (2014). Strong, lightweight, and recoverable three-dimensional ceramic nanolattices. Science.

[55] Wadley H.N., Fleck N.A., Evans A.G. (2003). Fabrication and structural performance of periodic cellular metal sandwich structures. Compos. Sci. Technol..

[56] Alzahrani M., Choi S.K., Rosen D.W. (2015). Design of truss-like cellular structures using relative density mapping method. Mater. Des..

[57] Tao W., Leu M.C. Design of lattice structure for additive manufacturing. Proceedings of the 2016 International Symposium on Flexible Automation (ISFA).

[58] Saleh Alghamdi S., John S., Roy Choudhury N., Dutta N.K. (2021). Additive Manufacturing of Polymer Materials: Progress, Promise and Challenges. Polymers.

[59] Giubilini A., Bondioli F., Messori M., Nyström G., Siqueira G. (2021). Advantages of Additive Manufacturing for Biomedical Applications of Polyhydroxyalkanoates. Bioengineering.

[60] Pryadko A., Surmeneva M.A., Surmenev R.A. (2021). Review of Hybrid Materials Based on Polyhydroxyalkanoates for Tissue Engineering Applications. Polymers.

[61] Ruban R., Rajashekhar V.S., Nivedha B., Mohit H., Sanjay M.R., Siengchin S., Khan M.A., Jappes J.T.W. (2022). Role of Additive Manufacturing in Biomedical Engineering. Innovations in Additive Manufacturing. Springer Tracts in Additive Manufacturing.

[62] Sheoran A.J., Kumar H., Arora P.K. (2020). Moona, GBio-Medical applications of Additive Manufacturing: A Review. Procedia Manuf..

[63] Kumar R., Kumar M., Chohan J. (2021). SThe role of additive manufacturing for biomedical applications: A critical review. J. Manuf. Processes.

[64] Popov V.V., Muller-Kamskii G., Kovalevsky A., Dzhenzhera G., Strokin E., Kolomiets A., Ramon J. (2018). Design and 3D-printing of titanium bone implants: Brief review of approach and clinical cases. Biomed. Eng. Lett..

[65] Shakibania S., Ghazanfari L., Raeeszadeh-Sarmazdeh M., Khakbiz M. (2021). Medical application of biomimetic 4D printing. Drug Dev. Ind. Pharm..

[66] Javaid M., Haleem A. (2019). 4D printing applications in medical field: A brief review. Clin. Epidemiol. Glob. Health.

[67] Agarwal T., Hann S.Y., Chiesa I., Cui H., Celikkin N., Micalizzi S., Barbetta A., Costantini M., Esworthy T., Zhang L.G. (2021). 4D printing in biomedical applications: Emerging trends and technologies. J. Mater. Chem. B.

[68] Zhou W., Qiao Z., Zare E.N., Huang J., Zheng X., Sun X., Shao M., Wang H., Wang X., Chen D. (2020). 4D-Printed Dynamic Materials in Biomedical Applications: Chemistry, Challenges, and Their Future Perspectives in the Clinical Sector. J. Med. Chem..

[69] Zheng X., Smith W., Jackson J., Moran B., Cui H., Chen D., Ye J., Fang N., Rodriguez N., Weisgraber T. (2016). Multiscale metallic metamaterials. Nat. Mater..

[70] Yang L., Cormier D., West H., Knowlson K. (2012). NonStochastic Ti-6Al-4V Foam Structure That Shows Negative Poisson’s Ratios. Mater. Sci. Eng. A.

[71] Yang L., Harrysson O., Cormier D., West H. (2012). Compressive Properties of Ti-6Al-4V Auxetic Mesh Structures Made by EBM Process. Acta Mater..

[72] Yang L., Harrysson O., Cormier D., West H. (2012). Modeling of the Uniaxial Compression of a 3D Periodic ReEntrant Honeycomb Structure. J. Mater. Sci..

[73] Cuan-Urquizo E., Álvarez-Trejo A., Robles Gil A., Tejada-Ortigoza V., Camposeco-Negrete C., Uribe-Lam E., Treviño-Quintanilla C.D. (2022). Effective Stiffness of Fused Deposition Modeling Infill Lattice Patterns Made of PLA-Wood Material. Polymers.

[74] Kladovasilakis N., Charalampous P., Tsongas K., Kostavelis I., Tzetzis D., Tzovaras D. (2021). Experimental and Computational Investigation of Lattice Sandwich Structures Constructed by Additive Manufacturing Technologies. J. Manuf. Mater. Process..

[75] RAENG (2013). Additive Manufacturing: Opportunities and Constraints.

[76] Wohler T. (2013). Wohler’s Report 2013-Additive Manufacturing and 3D Printing State of the Industry. Annual Worldwide Progress Report.

[77] Obi M.U., Pradel P., Sinclair M., Bibb R. (2022). A bibliometric analysis of research in design for additive manufacturing. Rapid Prototyp. J..

[78] Caviggioli F., Ughetto E. (2019). A bibliometric analysis of the research dealing with the impact of additive manufacturing on industry, business and society. Int. J. Prod. Econ..

[79] Patil A.K., Soni G. (2020). A State of The Art Bibliometric Analysis For Additive Manufacturing. Curr. Mater. Sci..

[80] Zhou Y., Tang Y., Hoff T., Garon M., Zhao F.Y. (2015). The Verification of the Mechanical Properties of Binder Jetting Manufactured Parts by Instrumented Indentation Testing. Procedia Manuf..

[81] Forster A.M. (2015). Materials Testing Standards for Additive Manufacturing of Polymer Materials: State of the Art and Standards Applicability.

[82] Mao M., He J., Li X., Zhang B., Lei Q., Liu Y., Li D. (2017). The Emerging Frontiers and Applications of High-Resolution 3D Printing. Micromachines.

[83] Bezek L.B., Cauchi M.P., de Vita R., Foerst J.R., Williams C.B. (2020). 3D Printing Tissue-Mimicking Materials for Realistic Transseptal Puncture Models. J. Mech. Behav. Biomed. Mater..

[84] Miyanaji H., Ma D., Atwater M.A., Darling K.A., Hammond V.H., Williams C.B. (2020). Binder Jetting Additive Manufacturing of Copper Foam Structures. Addit. Manuf..

[85] Herzberger J., Sirrine J.M., Williams C.B., Long T.E. (2019). Polymer Design for 3D Printing Elastomers: Recent Advances in Structure, Properties, and Printing. Prog. Polym. Sci..

[86] Sturm L.D., Albakri M.I., Tarazaga P.A., Williams C.B. (2019). In Situ Monitoring of Material Jetting Additive Manufacturing Process via Impedance Based Measurements. Addit. Manuf..

[87] Chatham C.A., Long T.E., Williams C.B. (2019). A Review of the Process Physics and Material Screening Methods for Polymer Powder Bed Fusion Additive Manufacturing. Prog. Polym. Sci..

[88] Moher D., Shamseer L., Clarke M., Ghersi D., Liberati A., Petticrew M., Shekelle P., Stewart L.A., Group P.-P., Altman D.G. (2015). Preferred reporting items for systematic review and meta-analysis protocols (PRISMA-P) 2015 statement. Syst. Rev..

[89] Martinez P., Al-Hussein M., Ahmad R. (2019). A scientometric analysis and critical review of computer vision applications for construction. Autom. Constr..

[90] Hood W.W., Wilson C.S. (2001). The literature of bibliometrics, scientometrics, and informetrics. Scientometrics.

[91] Jin Y., Li X., Campbell R.I., Ji S. (2018). Visualizing the hotspots and emerging trends of 3D printing through scientometrics. Rapid Prototyp. J..

[92] Jemghili R., Taleb A.A., Mansouri K. (2021). A bibliometric indicators analysis of additive manufacturing research trends from 2010 to 2020. Rapid Prototyp. J..

[93] Parvanda R., Kala P., Sharma V. (2021). Bibliometric Analysis-Based Review of Fused Deposition Modeling 3D Printing Method (1994–2020). 3D Printing and Additive Manufacturing.

[94] Dzogbewu T.C., Amoah N., Fianko S.K., Afrifa S., de Beer D. (2022). Additive manufacturing towards product production: A bibliometric analysis. Manuf. Rev..

[95] Perianes-Rodriguez A., Waltman L., Van Eck N.J. (2016). Constructing bibliometric networks: A comparison between full and fractional counting. J. Informetr..

[96] Granovsky Y.V. (2001). Is it possible to measure science? V. V. Nalimov’s research in scientometrics. Scientometrics.

[97] Park J.Y., Nagy Z. (2018). Data on the interaction between thermal comfort and building control research. Data Brief.

[98] Wu Z., Yang K., Lai X., Antwi-Afari M.F. (2020). A Scientometric Review of System Dynamics Applications in Construction Management Research. Sustainability.

[99] Soosaraei M., Khasseh A.A., Fakhar M., Hezarjaribi H.Z. (2018). A decade bibliometric analysis of global research on leishmaniasis in Web of Science database. Ann. Med. Surg..

[100] Cash-Gibson L., Rojas-Gualdrón D.F., Pericàs J.M., Benach J. (2018). Inequalities in global health inequalities research: A 50-year bibliometric analysis (1966–2015). PLoS ONE.

[101] Sweileh W.M., Al-Jabi S.W., Zyoud S.H., Sawalha A.F., Abu-Taha A.S. (2018). Global research output in antimicrobial resistance among uropathogens: A bibliometric analysis (2002–2016). J. Glob. Antimicrob. Resist..

[102] Krauskopf E. (2018). A bibiliometric analysis of the Journal of Infection and Public Health: 2008–2016. J. Infect. Public Health.

[103] Van Eck N.J., Waltman L. (2022). VOSviewer Manual: Manual for VOSviewer Version 1.6.18.

[104] Van Eck N.J., Waltman L., Lenz H.-J., Decker R. (2007). VOS: A new method for visualizing similarities between objects. Advances in Data Analysis: Proceedings of the 30th Annual Conference of the German Classification Society.

[105] Van Eck N.J., Waltman L. (2009). How to normalize cooccurrence data? An analysis of some well-known similarity measures. J. Am. Soc. Inf. Sci. Technol..

[106] Van Eck N.J., Waltman L. (2010). Software survey: VOSviewer, a computer program for bibliometric mapping. Scientometrics.

[107] Van Eck N.J., Waltman L. (2011). Text mining and visualization using VOSviewer. ISSI Newsl..

[108] Van Eck N.J., Waltman L., Ding Y., Rousseau R., Wolfram D. (2014). Visualizing bibliometric networks. Measuring Scholarly Impact: Methods and Practice.

[109] Van Eck N.J., Waltman L., Dekker R., Van den Berg J. (2010). A comparison of two techniques for bibliometric mapping: Multidimensional scaling and VOS. J. Am. Soc. Inf. Sci. Technol..

[110] Van Nunen K., Li J., Reniers G., Ponnet K. (2018). Bibliometric analysis of safety culture research. Saf. Sci..

[111] Waltman L., Van Eck N.J. (2013). A smart local moving algorithm for large-scale modularity-based community detection. Eur. Phys. J. B.

[112] Waltman L., Van Eck N.J., Noyons E.C.M. (2010). A unified approach to mapping and clustering of bibliometric networks. J. Informetr..

[113] Wood J., Khan G.F. (2015). International trade negotiation analysis: Network and semantic knowledge infrastructure. Scientometrics.

[114] Chandra Y. (2018). Mapping the evolution of entrepreneurship as a field of research (1990–2013): A scientometric analysis. PLoS ONE.

[115] Chen C. (2004). Searching for intellectual turning points: Progressive Knowledge Domain Visualization. Proc. Natl. Acad. Sci. USA.

[116] Chen C. (2006). CiteSpace II: Detecting and visualizing emerging trends and transient patterns in scientific literature. JASIST.

[117] Chen C. (2010). System and Method for Automatically Generating Systematic Reviews of a Scientific Field. U.S. Patent.

[118] Chen C. (2016). CiteSpace: A Practical Guide for Mapping Scientific Literature.

[119] Chen C. (2017). Science mapping: A systematic review of the literature. JDIS.

[120] Chen C. (2020). A Glimpse of the First Eight Months of the COVID-19 Literature on Microsoft Academic Graph. Front. Res. Metr. Anal..

[121] Chen C., Ibekwe-Sanjuan F., Hou J. (2010). The structure and dynamics of co-citation clusters: A multiple-perspective co-citation analysis. J. Am. Soc. Inf. Sci. Technol. (JASIST).

[122] Chen C., Song M. (2019). Visualizing a field of research: A methodology of systematic scientometric reviews. PLoS ONE.

[123] Scimago (2022). Scimago Journal & Country Rank. Scimango Lab. https://www.scimagojr.com/journalrank.php.

[124] WoS (2022). Web of Science Database. https://www-webofscience-com.ezproxy.lancs.ac.uk/wos/woscc/basic-search.

[125] Scopus (2022). SCOPUS Database. Elsevier B.V. https://www-scopus-com.ezproxy.lancs.ac.uk/search/form.uri?display=basic#basic.

[126] Clarivate (2022). Journal Citation Reports. https://jcr.clarivate.com/jcr/home.

[127] Clarivate (2022). Journal Citation Reports™ (JCR) Infographics: Make Better Informed, More Confident Decisions. https://clarivate.com/webofsciencegroup/web-of-science-journal-citation-reports-2021-infographic/.

[128] AlRyalat SA S., Malkawi L.W., Momani S.M. (2019). Comparing Bibliometric Analysis Using PubMed, Scopus, and Web of Science Databases. J. Vis. Exp..

[129] Zhang Y., Liu H., Kang S.C., Al-Hussein M. (2020). Virtual reality applications for the built environment: Research trends and opportunities. Autom. Constr..

[130] Kim M.J., Wang X., Love P.E.D., Li H., Kang S.C. (2013). Virtual reality for the built environment: A critical review of recent advances. J. Inf. Technol. Constr..

[131] Hirsch J.E. (2005). An Index to Quantify an Individual’s Scientific Research Output. Proc. Natl. Acad. Sci. USA.

[132] Kamdem J.P., Duarte A.E., Lima K.R.R., Rocha J.B.T., Hassan W., Barros L.M., Roeder T., Tsopmo A. (2019). Research Trends in Food Chemistry: A Bibliometric Review of its 40 Years Anniversary (1976–2016). Food Chem..

[133] Olawumi T.O., Chan D.W. (2018). A scientometric review of global research on sustainability and sustainable development. J. Clean. Prod..

[134] Zheng C., Yuan J., Zhu L., Zhang Y., Shao Q. (2020). From digital to sustainable: A scientometric review of smart city literature between 1990 and 2019. J. Clean. Prod..

[135] Montoya F.G., Montoya M.G., Gómez J., Manzano-Agugliaro F., Alameda-Hernández E. (2014). The research on energy in Spain: A scientometric approach. Renew. Sustain. Energy Rev..

[136] Pollack J., Adler D. (2015). Emergent trends and passing fads in project management research: A scientometric analysis of changes in the field. Int. J. Proj. Manag..

[137] Chen K., Wang J., Yu B., Wu H., Zhang J. (2021). Critical evaluation of construction and demolition waste and associated environmental impacts: A scientometric analysis. J. Clean. Prod..

[138] Yin X., Liu H., Chen Y., Al-Hussein M. (2019). Building information modelling for off-site construction: Review and future directions. Autom. Constr..

[139] Chen D., Bi B., Luo Z.H., Yang Y.W., Webber M., Finlayson B. (2018). A scientometric review of water research on the Yangtze River. Appl. Ecol. Environ. Res..

[140] Tariq S., Hu Z., Zayed T. (2021). Micro-electromechanical systems-based technologies for leak detection and localization in water supply networks: A bibliometric and systematic review. J. Clean. Prod..

[141] Fang J., Pan L., Gu Q.X., Juengpanich S., Zheng J.H., Tong C.H., Wang Z.Y., Nan J.J., Wang Y.F. (2020). Scientometric analysis of mTOR signaling pathway in liver disease. Ann Transl. Med..

[142] Oladinrin O., Gomis K., Jayantha W.M., Obi L., Rana M.Q. (2021). Scientometric Analysis of Global Scientific Literature on Aging in Place. Int. J. Environ. Res. Public Health.

[143] Wininger A.E., Fischer J.P., Likine E.F., Gudeman A.S., Brinker A.R., Ryu J., Maupin K.A., Lunsford S., Whipple E.C., Loder R.T. (2017). Bibliometric Analysis of Female Authorship Trends and Collaboration Dynamics Over JBMR’s 30-Year History. J. Bone Miner Res..

[144] Palmblad M., van Eck N.J. (2018). Bibliometric Analyses Reveal Patterns of Collaboration between ASMS Members. J. Am. Soc. Mass Spectrom..

[145] Yang L., Harrysson O., West H., Cormier D. (2013). A Comparison of Bending Properties for Cellular Core Sandwich Panels. Mater. Sci. Appl..

[146] Ladani L., Romano J., Brindley W., Burlatsky S. (2017). Effective liquid conductivity for improved simulation of thermal transport in laser beam melting powder bed technology. Addit. Manuf..

[147] Galati M., Iuliano L., Salmi A., Atzeni E. (2017). Modelling energy source and powder properties for the development of a thermal FE model of the EBM additive manufacturing process. Addit. Manuf..

[148] Hwang T., Woo Y.Y., Han S.W., Moon Y.H. (2018). Functionally graded properties in directed-energy-deposition titanium parts. Opt. Laser Technol..

[149] Bletzinger K.U., Ramm E. (2001). Structural optimization and form-finding of lightweight structures. Comput. Struct..

[150] Mostafa K.G., Momesso G.A., Li X., Nobes D.S., Qureshi A.J. (2021). Dual Graded Lattice Structures: Generation Framework and Mechanical Properties Characterization. Polymers.

[151] Mustafa S.S., Lazoglu I. (2021). A new model and direct slicer for lattice structures. Struct. Multidisc. Optim..

[152] Guerra Silva R., Torres M.J., Zahr Viñuela J., Zamora A.G. (2021). Manufacturing and Characterization of 3D Miniature Polymer Lattice Structures Using Fused Filament Fabrication. Polymers.

[153] Al-Ketan O., Lee D., Al-Rub R.K.A. (2021). Mechanical properties of additively-manufactured sheet-based gyroidal stochastic cellular materials. Addit. Manuf..

[154] Maskery I., Parry L.A., Padrao D., Hague R.J.M., Ashcroft I.A. (2022). FLatt Pack: A research-focussed lattice design program. Addit. Manuf..

[155] Saremian R., Badrossamay M., Foroozmehr E., Kadkhodaei M., Forooghi F. (2021). Experimental and numerical investigation on lattice structures fabricated by selective laser melting process under quasi-static and dynamic loadings. Int. J. Adv. Manuf. Technol..

[156] Habib F.N., Iovenitti P., Masood S.H., Nikzad M. (2018). Fabrication of polymeric lattice structures for optimum energy absorption using Multi Jet Fusion technology. Mater. Des..

[157] Woodward I.R., Fromen C.A. (2021). Scalable, process-oriented beam lattices: Generation, characterization, and compensation for open cellular structures. Addit. Manuf..

[158] Hanks B., Frecker M. 3D Additive Lattice Topology Optimization: A Unit Cell Design Approach. Proceedings of the ASME 2020 International Design Engineering Technical Conferences and Computers and Information in Engineering Conference, 46th Design Automation Conference (DAC).

[159] Hanks B., Frecker M. Lattice Structure Design for Additive Manufacturing: Unit Cell Topology Optimization. Proceedings of the ASME 2019 International Design Engineering Technical Conferences and Computers and Information in Engineering Conference, 45th Design Automation Conference.

[160] Sienkiewicz J., Płatek P., Jiang F., Sun X., Rusinek A. (2020). Investigations on the Mechanical Response of Gradient Lattice Structures Manufactured via SLM. Metals.

[161] Abusabir A., Khan M.A., Asif M., Khan K.A. (2022). Effect of Architected Structural Members on the Viscoelastic Response of 3D Printed Simple Cubic Lattice Structures. Polymers.

[162] McConaha M., Anand S. Design of Stochastic Lattice Structures for Additive Manufacturing. Proceedings of the ASME 2020 15th International Manufacturing Science and Engineering Conference. Volume 1: Additive Manufacturing, Advanced Materials Manufacturing; Biomanufacturing, Life Cycle Engineering; Manufacturing Equipment and Automation.

[163] Al-Ketan O., Al-Rub R.K.A. (2020). MSLattice: A free software for generating uniform and graded lattices based on triply periodic minimal surfaces. Mater. Des. Processing Commun. (MDPC).

[164] Riva L., Ginestra P.S., Ceretti E. (2021). Mechanical characterization and properties of laser-based powder bed–fused lattice structures: A review. Int. J. Adv. Manuf. Technol..

[165] Nazir A., Abate K.M., Kumar A., Jeng J.Y. (2019). A state-of-the-art review on types, design, optimization, and additive manufacturing of cellular structures. Int. J. Adv. Manuf. Technol..

[166] Kumar A., Collini L., Daurel A., Jeng J. (2020). Design and additive manufacturing of closed cells from supportless lattice structure. Addit. Manuf..

[167] Jamshidinia M., Kong F., Kovacevic R. (2013). The Numerical Modeling of Fatigue Properties of a Bio-Compatible Dental Implant Produced by Electron Beam Melting^®^ (EBM). Proceedings of the Conference: Twenty Forth Annual International Solid Freeform Fabrication Symposium. https://www.researchgate.net/publication/270822365_The_numerical_modeling_of_fatigue_properties_of_a_bio-compatible_dental_implant_produced_by_Electron_Beam_MeltingR_EBM.

[168] Bacciaglia A., Ceruti A., Liverani A. (2020). Proposal of a standard for 2D representation of bio-inspired lightweight lattice structures in drawings. Proc. Inst. Mech. Eng. Part C J. Mech. Eng. Sci..

[169] ISO/ASTM (2015). International standard ISO/ASTM 52900 additive manufacturing—General principles—Terminology. Int. Organ. Stand..

[170] Rendeki S., Nagy B., Bene M., Pentek A., Toth L., Szanto Z., Told R., Maroti P. (2020). An Overview on Personal Protective Equipment (PPE) Fabricated with Additive Manufacturing Technologies in the Era of COVID-19 Pandemic. Polymers.

[171] Tarfaoui M., Nachtane M., Goda I., Qureshi Y., Benyahia H. (2020). Additive manufacturing in fighting against novel coronavirus COVID-19. Int. J. Adv. Manuf. Technol..

[172] Radfar P., Bazaz S.R., Mirakhorli F., Warkiani M.E. (2021). The role of 3D printing in the fight against COVID-19 outbreak. J. 3D Print. Med..

[173] Agarwal R. (2022). The personal protective equipment fabricated via 3D printing technology during COVID-19. Ann. 3D Print. Med..

[174] Equbal A., Akhter S., Sood A.K., Equbal I. (2021). The usefulness of additive manufacturing (AM) in COVID-19. Ann. 3D Print. Med..

[175] Longhitano G.A., Nunes G.B., Candido G., da Silva J.V.L. (2021). The role of 3D printing during COVID-19 pandemic: A review. Prog. Addit. Manuf..

[176] Zuniga J.M., Cortes A. (2020). The role of additive manufacturing and antimicrobial polymers in the COVID-19 pandemic. Expert Rev. Med. Devices.

[177] Kunkel M.E., Vasques M.T., Perfeito J.A.J., Zambrana N.R.M., Bina T.D.S., Passoni L.H.D.M., Ribeiro T.V., Rodrigues S.M.S., Castro R.O.M.D., Ota N.H. (2020). Mass-production and distribution of medical face shields using additive manufacturing and injection molding process for healthcare system support during COVID-19 pandemic in Brazil. Res. Sq..

[178] Swennen G.R.J., Pottel L., Haers P.E. (2020). Custom-made 3D-printed face masks in case of pandemic crisis situations with a lack of commercially available FFP2/3 masks. Int. J. Oral. Maxillofac. Surg..

[179] Erickson M.M., Richardson E.S., Hernandez N.M., Bobbert II D.W., Gall K., Fearis P. (2020). Helmet modification to PPE with 3D printing during the COVID-19 pandemic at Duke University Medical Center: A novel technique. J. Arthroplast..

[180] Neijhoft J., Viertmann T., Meier S., Söhling N., Wicker S., Henrich D., Marzi I. (2020). Manufacturing and supply of face shields in hospital operation in case of unclear and confirmed COVID-19 infection status of patients. Eur. J. Trauma Emerg. Surg..

[181] Belhouideg S. (2020). Impact of 3D printed medical equipment on the management of the Covid19 pandemic. Int. J. Health Plann. Manag..

[182] Colorado H.A., Mendoza D.E., Lin H., Gutierrez-Velasquez E. (2022). Additive manufacturing against the Covid-19 pandemic: A technological model for the adaptability and networking. J. Mater. Res. Technol..

[183] Amin D., Nguyen N., Roser S.M., Abramowicz S. (2020). 3D printing of face shields during COVID-19 pandemic: A technical note. J. Oral. Maxillofac. Surg..

[184] Helman S.N., Soriano R.M., Tomov M.L., Serpooshan V., Levy J.M., Pradilla G., Solares C.A. (2020). Ventilated upper airway endoscopic endonasal procedure mask: Surgical safety in the COVID-19 era. Oper. Neurosurg..

[185] Kalyaev V., Salimon A.I., Korsunsky A.M. (2020). Fast mass-production of medical safety shields under COVID-19 quarantine: Optimizing the use of university fabrication facilities and volunteer labor. Int. J. Environ. Res. Public Health.

[186] Tino R., Moore R., Antoline S., Ravi P., Wake N., Ionita C.N., Morris J.M., Decker S.J., Sheikh A., Rybicki F.J. (2020). COVID-19 and the role of 3D printing in medicine. 3D Print. Med..

[187] Das H., Patowary A. (2020). Uses of 3D Printing for Production of Ppe for Covid 19 like situations: Scope and future. Am. J. Prev. Med. Public Health.

[188] Larrañeta E., Dominguez-Robles J., Lamprou D.A. (2020). Additive Manufacturing can assist in the fight against COVID-19 and other pandemics and impact on the global supply chain. 3D Print. Addit. Manuf..

[189] Novak J.I., Loy J. (2020). A quantitative analysis of 3D printed face shields and masks during COVID-19. Emerald Open Res..

[190] Sinha M.S., Bourgeois F.T., Sorger P.K. (2020). Personal protective equipment for COVID-19: Distributed fabrication and additive manufacturing. Am. J. Public Health.

